# Atomic Regulation of PGM Electrocatalysts for the Oxygen Reduction Reaction

**DOI:** 10.3389/fchem.2021.699861

**Published:** 2021-07-06

**Authors:** Menghao Wu, Changli Chen, Yizhou Zhao, Enbo Zhu, Yujing Li

**Affiliations:** Beijing Key Laboratory of Construction Tailorable Advanced Functional Materials and Green Applications, Experimental Center of Advanced Materials, School of Materials Science and Engineering, Beijing Institute of Technology, Beijing, China

**Keywords:** electrocatalysts, oxygen reduction reaction, Platinum, Palladium, atomic regulation, structure engineering

## Abstract

With the increasing enthusiasm for the hydrogen economy and zero-emission fuel cell technologies, intensive efforts have been dedicated to the development of high-performance electrocatalytic materials for the cathodic oxygen reduction reaction (ORR). Some major fundamental breakthroughs have been made in the past few years. Therefore, reviewing the most recent development of platinum-group-metal (PGM) ORR electrocatalysts is of great significance to pushing it forward. It is known that the ORR on the fuel cell electrode is a heterogeneous reaction occurring at the solid/liquid interface, wherein the electron reduces the oxygen along with species in the electrolyte. Therefore, the ORR kinetic is in close correlation with the electronic density of states and wave function, which are dominated by the localized atomic structure including the atomic distance and coordination number (CN). In this review, the recent development in the regulation over the localized state on the catalyst surface is narrowed down to the following structural factors whereby the corresponding strategies include: the crystallographic facet engineering, phase engineering, strain engineering, and defect engineering. Although these strategies show distinctive features, they are not entirely independent, because they all correlate with the atomic local structure. This review will be mainly divided into four parts with critical analyses and comparisons of breakthroughs. Meanwhile, each part is described with some more specific techniques as a methodological guideline. It is hoped that the review will enhance an insightful understanding on PGM catalysts of ORR with a visionary outlook.

## Introduction

The social and scientific concerns on the use of non-renewable fossil fuels and their environmental influences have long been the driving force toward energy evolution. Compared with the traditional energy conversion devices with efficiency normally lower than 60%, electrochemistry-based technologies show the merit of higher energy conversion efficiency. Renewable energy sources such as hydrogen, alcohols, and other biomass can be directly converted into electricity with a theoretical efficiency of 85–90% through highly efficient fuel cell devices (Luo and Guo, 2017; Seh et al., 2017; Stamenkovic et al., 2017).

**FIGURE 1:**
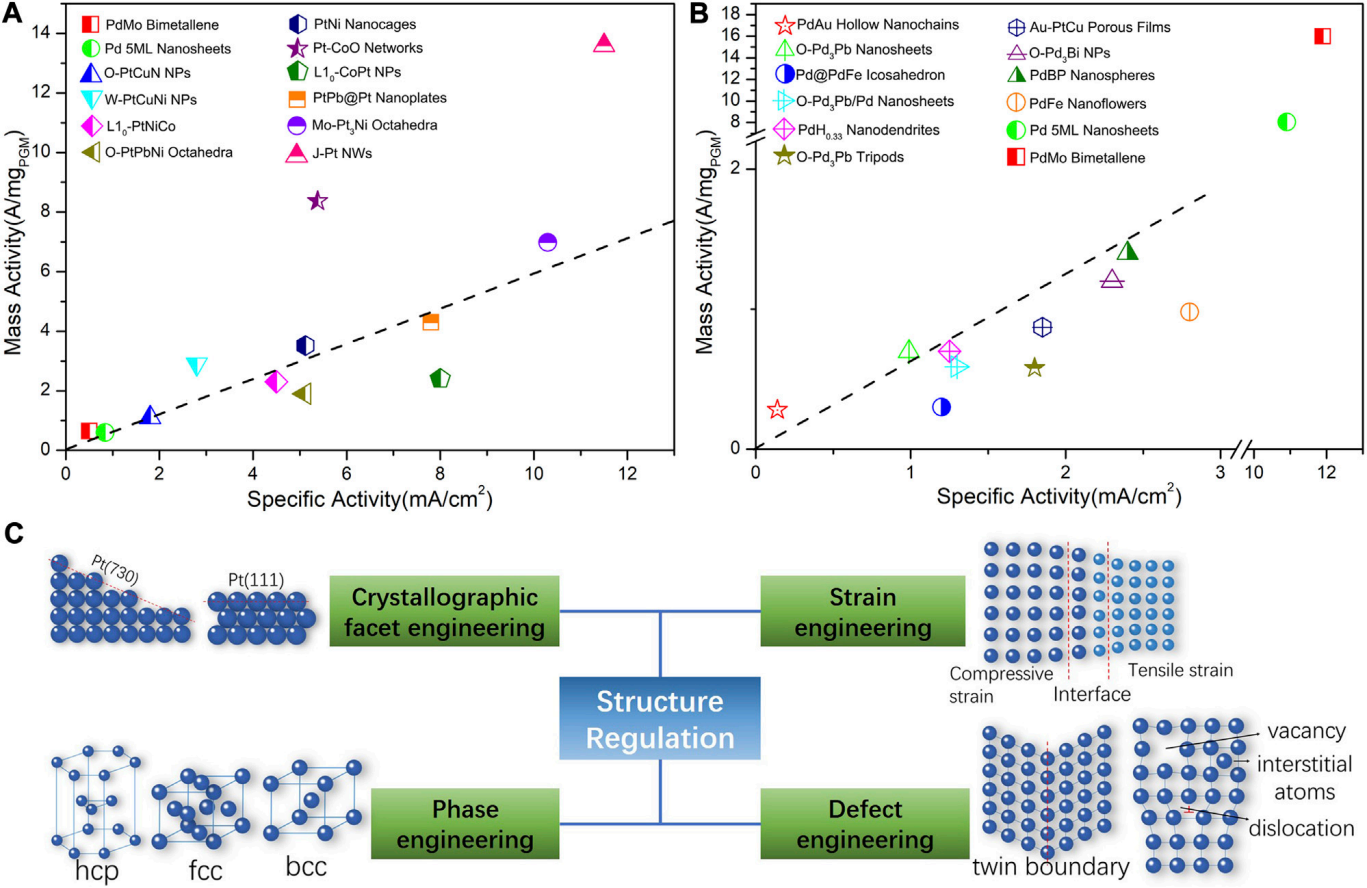
Current development of platinum-group-metal (PGM)-based nanocatalysts for ORR by structure regulation. **(A)** Mass activity (MA) and specific activity (SA) of ORR in 0.1 M HClO_4_. PdMo Bimetallene (Luo et al., 2019); Pd 5 ML Nanosheets (Wang et al., 2019a); O-PtCuN NPs (Zhao et al., 2020); Porous W-PtCuNi NPs (Tu et al., 2019); L10-PtNiCo (Wang et al., 2019c); O-PtPbNi Octahedra (Bu et al., 2017); PtNi Nanocages (Tian et al., 2019); Pt-CoO Networks (Sievers et al., 2020); L_10_-CoPt NPs (Li et al., 2019a); PtPb@Pt Nanoplates (Bu et al., 2016b); Mo-Pt_3_Ni Octahedra (Huang et al., 2015); J-Pt NWs (Li et al., 2016). The dashed line represents a suppositional catalyst with electrochemical surface area (ECSA) of 60 m^2^/g_PGM_. **(B)** Mass activity and specific activity of ORR in 0.1 M KOH. PdAu Hollow Nanochains (Jiao et al., 2020); O-Pd_3_Pb Nanosheets (Bu et al., 2018b); Pd@PdFe Icosahedron (Li et al., 2020c); O-Pd_3_Pb/Pd Nanosheets (Tang et al., 2019); PdH_0.33_ Nanodendrites (Wang et al., 2019b); O-Pd_3_Pb Tripods (Bu et al., 2018a); Au-PtCu Porous Films (Xie et al., 2020); O-Pd_3_Bi NPs (Sun et al., 2019); PdBP Nanospheres (Lv et al., 2019); PdFe Nanoflowers (Lian et al., 2018); Pd 5 ML Nanosheets (Wang et al., 2019a); PdMo Bimetallene (Luo et al., 2019). The dashed line represents a suppositional catalyst with ECSA of 60 m^2^/g_PGM_. **(C)** Strategies to optimize the catalytic activities of solid catalyst from the structure regulation. Vacancy and interstitial atoms which would not appear on the surface, as well as the edge dislocation that are mainly located in the bulk and will not influence the surface chemistry.

Oxygen reduction reaction (ORR) serves as the cathode half-reaction of some fuel cell type energy conversion devices such as proton exchange membrane fuel cells (PEMFCs), anion exchange membrane fuel cells (AEMFCs), and metal-air secondary batteries, etc. The intrinsic ORR kinetics could be order-of-magnitude lower than that of the anode reaction such as the hydrogen oxidation reaction (HOR), leading to an excessive reaction overpotential that reduces the output voltage and the output power of the fuel cells. As a result, expediting the ORR kinetics and reducing the overpotential is critical to the development of high-performance cathode electrocatalysts. At the operation condition, the ORR occurs at the solid/electrolyte interface within a heterogeneous regime. At a certain potential, the electron can reduce the oxygen along with species in the electrolyte. Each intermediate species from the corresponding sub-reaction step has its unique sensitivity to the catalytic structure, which will influence the micro-kinetics and the resulting mechanism. From the fundamental level, the binding energy of oxygen species in the adsorption-desorption process plays a central role and dominates the ORR kinetics. As a commonly accepted rule, the Sabatier principle demonstrates that the ORR activity correlates to the oxygen binding energy following a volcanic plot, indicating that the Pt has the almost perfect oxygen binding energy toward the ORR (Stamenkovic et al., 2017). It is common or rationale to optimize the binding energy toward the top of the volcanic plot to achieve the best activity. The modulation of adsorption energy relies on the adjustment of the surface electronic structure of the catalyst surface, more technically by tuning the localized surface atomic structure and hence accelerates the ORR kinetics. Therefore, the local atomic structure and the resulting electronic structure bridge the oxygen binding energy and catalytic activity. As shown in Figures 1A,B, great progress has been made in the development of ORR electrocatalysts in acidic and alkaline electrolyte. Among them, most success is achieved by adjusting the structure of the electrocatalysts (Wang et al., 2021).

To a certain extent, the completion of a catalytic process generally involves the in-plane atomic arrangement and inter-plane atomic stacking across 1–3 atomic layers at the top-surface and sub-surfaces. Therefore, due to their surface-sensitive characteristics, the rational design of catalysts should not be limited to specific nano-morphology or nanostructures. Instead, the focus should be placed on the local atomic structure at the surface. Generally, it is considered that Pt atoms serve as the catalytically active center of ORR (Calle-Vallejo et al., 2015). The Pt atomic sites and the surrounding atoms form isolated electronic environment namely the localized state. It has been recognized that excellent ORR intrinsic activity requires a proper electronic density of states and wave function, which are determined by the localized atomic structure. The catalysts surface structure strongly affects the ORR kinetics through the aforementioned correlation. More specifically, in addition to the atomic composition, the atomic arrangement at the surface and near-surface has a strong influence on the surface electronic structure. Therefore, at the sub-nanometer scale or atomic level, modulating the atomic distance and atomic coordination number (CN) are technically achievable methodologies to directly affect the localized state and hence to improve the ORR activity (Calle-Vallejo et al., 2015). The above logic has narrowed down to the surface atomic distance and CN. Nonetheless, the methodology to manipulate these two factors are diversified and complicated. Combining theoretical and exemplary investigation, the adjustment of the localized surface state of the solid catalysts can be achieved through four strategies: crystallographic facet engineering, phase engineering, strain engineering, and defect engineering, as illustrated in Figure 1C. Different from previous reviews which are focused on the design of specific nano-structures, this mini review will attempt to correlate the most recent breakthroughs to some underlying structural factors spanning across the length scales from micrometer, nanometer and even down to sub-nanometer, which can be narrowed down to the crystallographic facet, crystal phase, strain, and defect at a more critical level.

## Crystallographic Facet Engineering

### Fundamentals of Faceting

**FIGURE 2:**
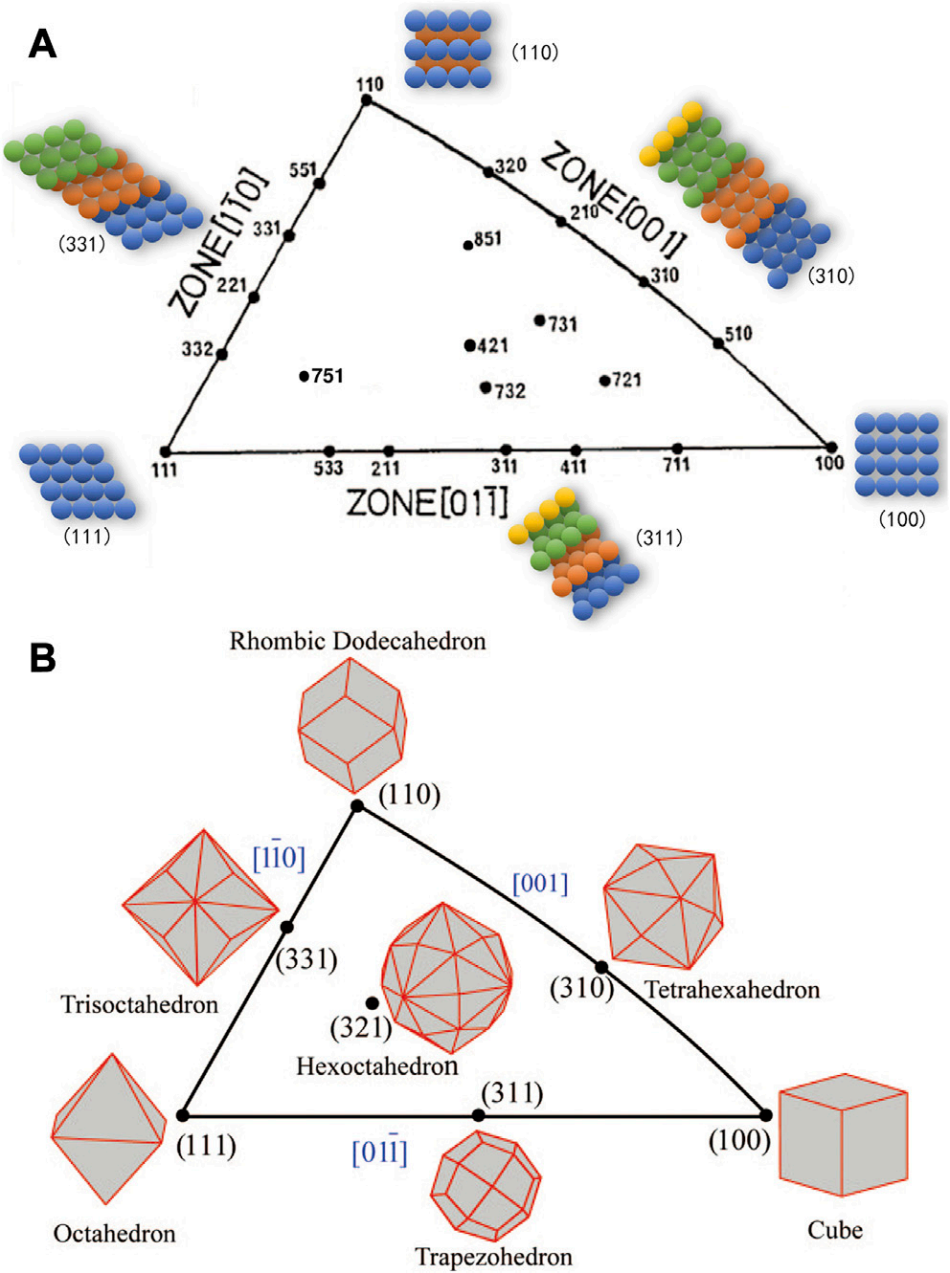
The unit stereographic triangle of fcc single crystal. **(A)** Crystallographic facets illustration and crystallographic facets diagram of fcc single crystal. **(B)** Schematic diagram of single crystal surrounded by some crystallographic facets. **(B)** Reproduced with permission (Tian et al., 2008). Copyright 2008, American Chemical Society.

Nanocatalysts, typical of nanocrystalline particles, are normally surrounded by favored crystallographic facets. The unique arrangement of surface atoms on different facets, e.g., the step densities, coordination numbers, and kinked atoms, will have a significant impact on ORR activity, resulting in the crystallographic facets structure effect. The plane passing through any three lattice points in the crystal is known as the crystallographic facet, with a well-defined Miller Index (hkl). Since most noble metals possess the face-centered cubic (fcc) lattice, the unit stereographic triangle projection of the fcc metal single crystal is used to illustrate the characteristics of various crystallographic facets in spherical polar coordinates, as shown in Figure 2A (Tian et al., 2008). The three vertices represent the basic singular crystallographic facets, whereas the lines and the inside region represent the high-index facets, also known as the stepped facets or vicinal facets (Tian et al., 2008). On the surface, two crystallographic facets will intersect at the edge. When the intersecting edges of the crystallographic facets are parallel to each other, all these crystallographic facets form a crystal zone. As the diagram suggests, the three edges of the unit triangle represent the three basic crystal zones. Each point at the edge represents a family of crystallographic facets parallel to the corresponding crystal zone axis.

As shown in Figure 2B, the three vertices of the triangle correspond to (111), (100), and (110) crystallographic facets respectively, whereby the polyhedra enclosed by these facets could be octahedrons, cubes, and rhombic dodecahedrons, with the coordination number of surface atoms to be 9, 8, and 7, respectively. As examples, the polyhedrons enclosed by the facets on the side line could be surrounded by 24 high-index crystallographic facets, including the tetrahexahedra (THH) surrounded by (hk0) (h > k > 0), trapezohedron by (hkk) (h > k > 0), and trisoctahedra by (hhk) (h > k > 0), with the corresponding surface atomic coordination numbers to be 6, 7, and 7, respectively. The polyhedron enclosed by facets inside the triangle is a hexaoctahedron (HOH) surrounded by (hkl) (h > k > l ≥ 1) crystallographic facets, which exposes 48 facets. The crystallographic facets (111) and (100) show the least steps, wherein the atoms are closely packed.

As a contrast, the high-index crystallographic facets, also known as vicinal surface or non-singular surface, have contain denser steps or kinked atoms, leading to lower coordination number (Yu et al., 2019). The low-coordination surface atoms possess abundant dangling bonds, which enables an easier interaction with the adsorbing molecules and hence turns into catalytically active centers and improves the catalytic activity. Due to the higher density of atoms with low coordination number, the high-index facets commonly display higher surface energy than the low-index counterparts, leading to thermodynamic instability.

### Facet-Controlled Nanocrystal Synthesis

**FIGURE 3:**
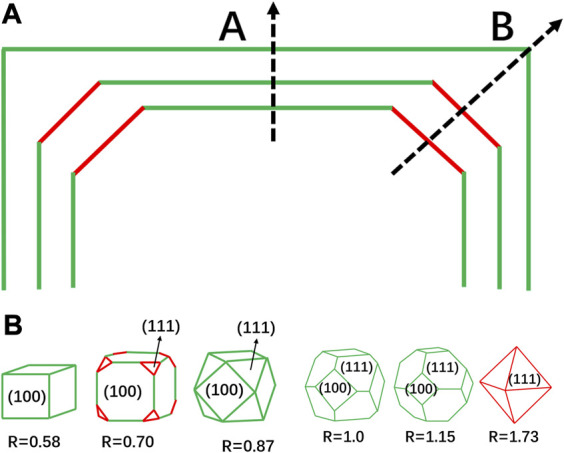
Facets control of metal nanocrystals. **(A)** The growth rate along the B crystallographic direction is higher than that along the A direction, leading to the disappearance of the B crystallographic facet. **(B)** Different growth rate ratios will lead to differences in the final crystallographic facet, R is the ratio of <100> and <111> crystal growth rates.

The greatest challenge for the catalysts with high index crystallographic facets lies in the synthesis. The crystallization of metallic nanocrystals can typically be divided into two stages: nucleation and growth. Generally, crystal nucleation occurs as the precursor reaches a critical saturation point (Yu et al., 2011). Once the nuclei size surpasses the critical size, the nuclei will eventually grow into crystals. The morphology of the nanocrystal is closely related to the surface energy of different crystallographic facets. The growth rate normal to the crystallographic facets with higher surface energy is faster, so these high-index crystallographic facets tend to disappear. As illustrated in Figure 3, if Facet B possesses higher surface energy, the crystals will show a faster growth rate along the crystallographic direction normal to Facet B, and eventually have the B surface eliminated. Besides, the surface energy of the typical crystallographic facets increases in the order (111) < (100) < (110) < (hkl) (h > k > 1) for the fcc metals. As example, for the two low-index crystallographic facets (100) and (111), the varied relative growth rates of the two facets will eventually form various crystal morphologies, as shown in Figure 3B, wherein the R represents the ratio of crystal growth rates of <100> and <111> (Tian et al., 2008).

Modulating the surface energy is the key to the control of the nanocrystal morphology. Some organic molecules can preferentially bind to particular crystallographic facets, modulate the surface energy and hence influence the morphology (Huang et al., 2019). Therefore, specific surfactant molecules can be employed to adjust the surface energy for the morphology-targeted synthesis of nanocrystals. At a high reduction rate of the metal precursor, the seed crystals generally grow into single crystals, whereas the low reduction rate usually generates multiple-twin seeds, wherein the twin region is considered to be a highly catalytically active structure. Besides, other factors such as precursor, reducing agent, and reaction temperature will influence the final morphology (Tian et al., 2007; Yu et al., 2019). There are generally two ways to obtain nano-catalysts with high-index crystallographic facets. One strategy relies on the regulation over the surface energy, whereby the addition of organic surface capping agents is the mostly used method. However, the organic ligands are often difficult to be removed, which may be detrimental to the catalytic sites. Mirkin et al. reduced the surface energy by alloying with other elements, followed by the dealloying to achieve nanocrystals with high index crystallographic facets (Huang et al., 2019). They used trace amounts of Sb, Bi, Pb, and Te as auxiliary elements to alloy with Pt and obtained THH particles exposing high-index crystallographic facets. These trace elements could be removed by annealing as they have lower melting points. Furthermore, the approach can be successfully extended to THH nanoparticles (NPs) of Pd, Rh, Ni, and Co. Another strategy involves controling over the reaction kinetics. It is known that crystal growth includes three processes: precursor reduction, atomic diffusion, and atomic deposition. The selection of reducing agents, including the concentration of reducing agents, along with the seed concentration, temperature control, and injection rate, can be employed to control the crystal growth rate at various stages (Zhou et al., 2011; Huang et al., 2019).

### General Effect of Facet Engineering

**FIGURE 4:**
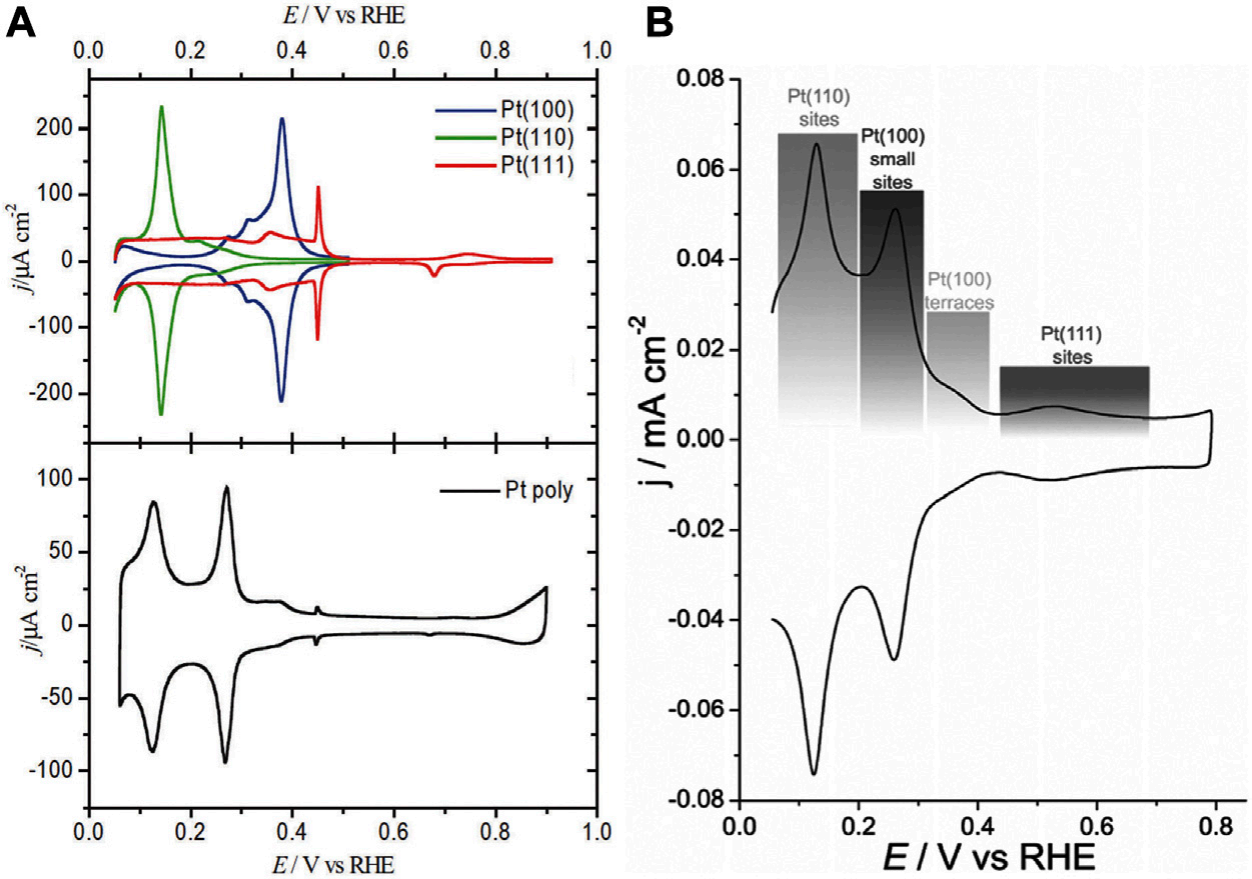
The cyclic voltammetry of the surface of crystalline Pt film in the 0.5 M sulfuric acid electrolyte at the sweep rate at 50 mV/s. **(A)** Single crystalline Pt. **(B)** Polycrystalline Pt. **(A,B)** Reproduced with permission (Gómez-Marín et al., 2014; Strasser et al., 2018). Copyright 2018, Royal Society of Chemistry.

The High-Resolution Transmission Electron Microscopy (HRTEM) was used previously to investigate the crystallographic facets exposure of crystalline materials by quantifying the atomic distance. For the bulk single crystals, the crystallographic facets can usually be discriminated through the electrochemical voltammetric technique, owing to the unique underpotential deposition and stripping response of H, which is highly sensitive to the surface structure (Vidal-Iglesias et al., 2012; Gómez-Marín et al., 2014). Figure 4 illustrates the typical electrochemical characterization of the three basic crystallographic facets of Pt by the cyclic voltammetry in 0.5 M sulfuric acid at 50 mV/s. It can also be indicated that it is possible to identify the exposed crystallographic facets on the surface of polycrystalline Pt by the voltammetric technique (Kuzume et al., 2007).

The characteristics of cyclic voltammetry (CV) can usually reflect the adsorption/desorption behavior of hydrogen on the crystal surface with a specific atomic arrangement structure, which can occur at a more positive potential than reversible hydrogen equilibrium potential (RHE) and has become the criterion for the determination of Pt facet at the solid-liquid interface. Similarly, the oxygen adsorption/desorption has also become the mostly accepted probe reactions.

As shown in Figure 4A, the desorption curve of upd-H varies with the structure of the crystallographic facets. The crystallographic facet of (100) shows an obvious characteristic peak near 0.31 V vs. RHE. For (110) crystallographic facet, the characteristic upd-H desorption peak appears at 0.17 V vs. RHE (Strasser et al., 2018).

The charge can be obtained by integrating H desorption current in CV curve:

$$\text{Q}\left(\text{E}\right)=\frac{1}{\text{ν}}\int_{{\text{E}}_{1}}^{\text{E}}\left[\text{j}\left(\text{u}\right)-{\text{j}}_{\text{dl}}\right]\text{du}$$

wherein, E and E_1_ are upper and lower limits of hydrogen desorption potential, dl represents double-layer capacitance and v stands for adsorption charge density. The charges induced by H adsorption on Pt (111), Pt (100), and Pt (110) are determined to be 240, 205, and 220 C/cm^2^, respectively (Tian et al., 2018) For Pt (111) and Pt (100), the values are close to the theoretical calculation value, meaning that one Pt atom active site adsorbs one H atom (full monolayer adsorption). It indicates that (111) and (100) crystallographic facets maintain atomic arrangement of (1 × 1) (Zhou et al., 2011) For Pt (110), the value is 1.5 times of the theoretical value (147 C/cm^2^), corresponding to an atomic arrangement of (1 × 2) due to surface reconstruction during the CV process (Zhou et al., 2011) Similarly, hydrogen desorption curves can be used to detect other metals that adsorb hydrogen, such as Ir, Rh. However, for Pd, the peaks originating from the absorption and release of H are overlapping with hydrogen absorption and desorption current. Therefore, the O desorption is usually used as a probe to detect the microstructure of the Pd surface and calculate the active area.

For polycrystalline Pt, the surface is usually considered to be a mixture of three basic crystallographic facets, with an average surface atomic density of 1.31 × 1,015/cm^2^. Therefore, the adsorption charge density of 210 C/cm^2^ can generally be used as a reference value (Tian et al., 2008; Xiao et al., 2020). For Pt (111) crystallographic facet, when the potential is lower than 0.05 V RHE, the H evolution current will arise, leading to a detection of only 2/3 monolayer H coverage above the lower limit of H desorption current of 0.05 V (RHE). Therefore, it will interference with the precise quantification of Pt (111) active surface area (Zhou et al., 2011).

As shown in Figure 4B, there are different current peaks in the H desorption region, indicating the existence of different hydrogen adsorption sites. The positive potential proved to be a strong H adsorption peak, which had strong binding energy with Pt atoms, such as Pt (111) sites (Kim et al., 2019a). Correspondingly, a negative potential corresponds to a weak Pt adsorption peak, such as Pt (110) sites. However, for most Pt catalysts, these peak distinctions may not be obvious due to the complex electrochemical environment. Even on the surface of a single crystal electrode, there were still multiple hydrogen adsorption current peaks, indicating the existence of different hydrogen adsorption sites and complex reactivity. In addition, anions also have a great influence on the cyclic voltammetric profile, which is due to the different adsorption structures and strength of anions on the electrode surface (Chen et al., 2010).

**FIGURE 5:**
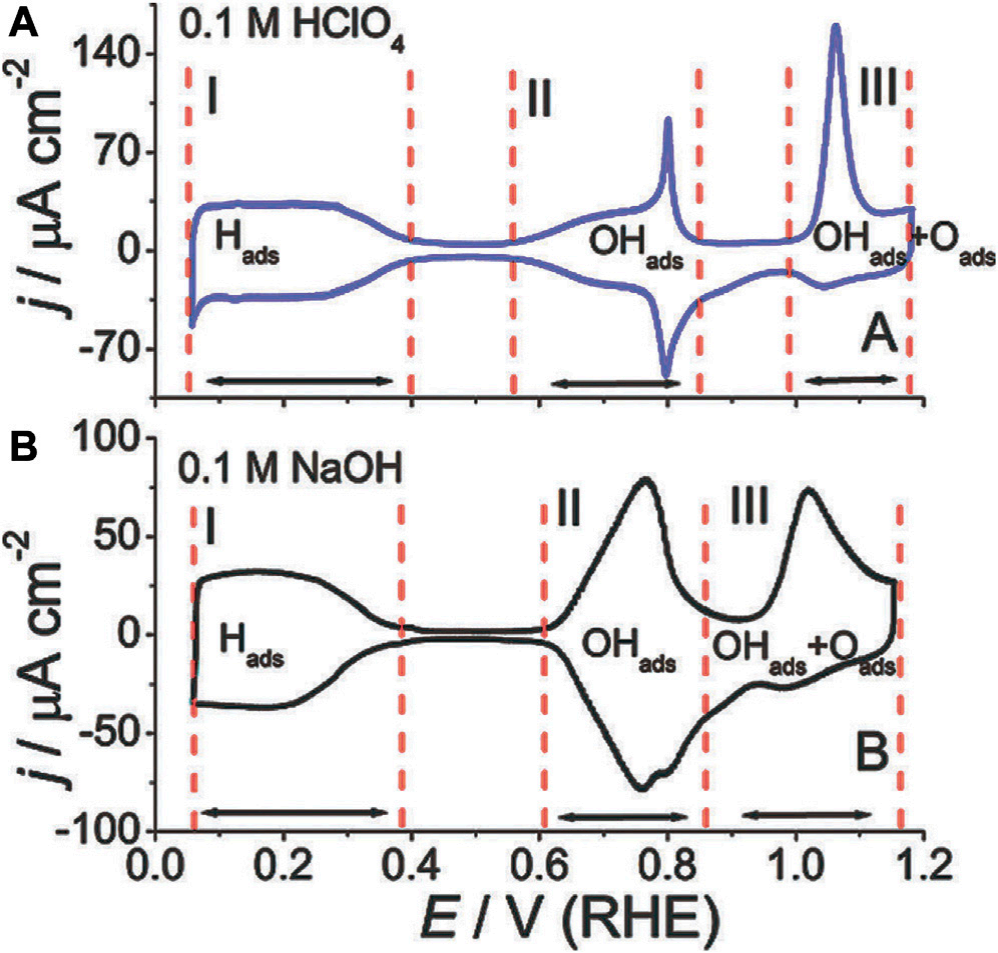
**(A,B)** CV curves of single crystalline Pt in different electrolytes. Reproduced with permission (Gómez-Marín et al., 2014). Copyright 2014, Royal Society of Chemistry.

Feliu et al. investigated the ORR kinetics on the Pt single crystalline surface (Vidal-Iglesias et al., 2012; Gómez-Marín et al., 2014). Theoretical calculations revealed that the atomic arrangement on exposed facets affects the adsorption energy of O-related species, which in turn influenced the activation energy of the target reactions (Kuzume et al., 2007). As shown in Figure 5, experimental tests showed that the ORR kinetics were not only seriously affected by the exposed crystallographic facets, but also by the structure of the adsorption layer determined by the nature of the electrolyte. More specifically, the ionic species from the electrolyte would lead to distinct coverages of OH_ads_ and O_ads_, which may further affect the adsorption process of surface oxygen and hence the activation energy of the subsequent reactions. In the perchloric acid electrolyte, the ORR kinetics increases in the order of Pt (100) ≪ Pt (111) < Pt (110). For Pt (111), in the sulfuric acid electrolyte, there is a group of butterfly peaks near 0.45 V RHE, as shown in Figure 5A. It is due to the adsorption structure changing of sulfate. But in the perchloric acid electrolyte, there are no peaks, as shown in Figure 5A. It demonstrates the influence of electrolyte types on active sites. In Figure 5, Pt (111) was used as a model system to demonstrate the different behaviors in adsorbed species in various environments.

**FIGURE 6:**
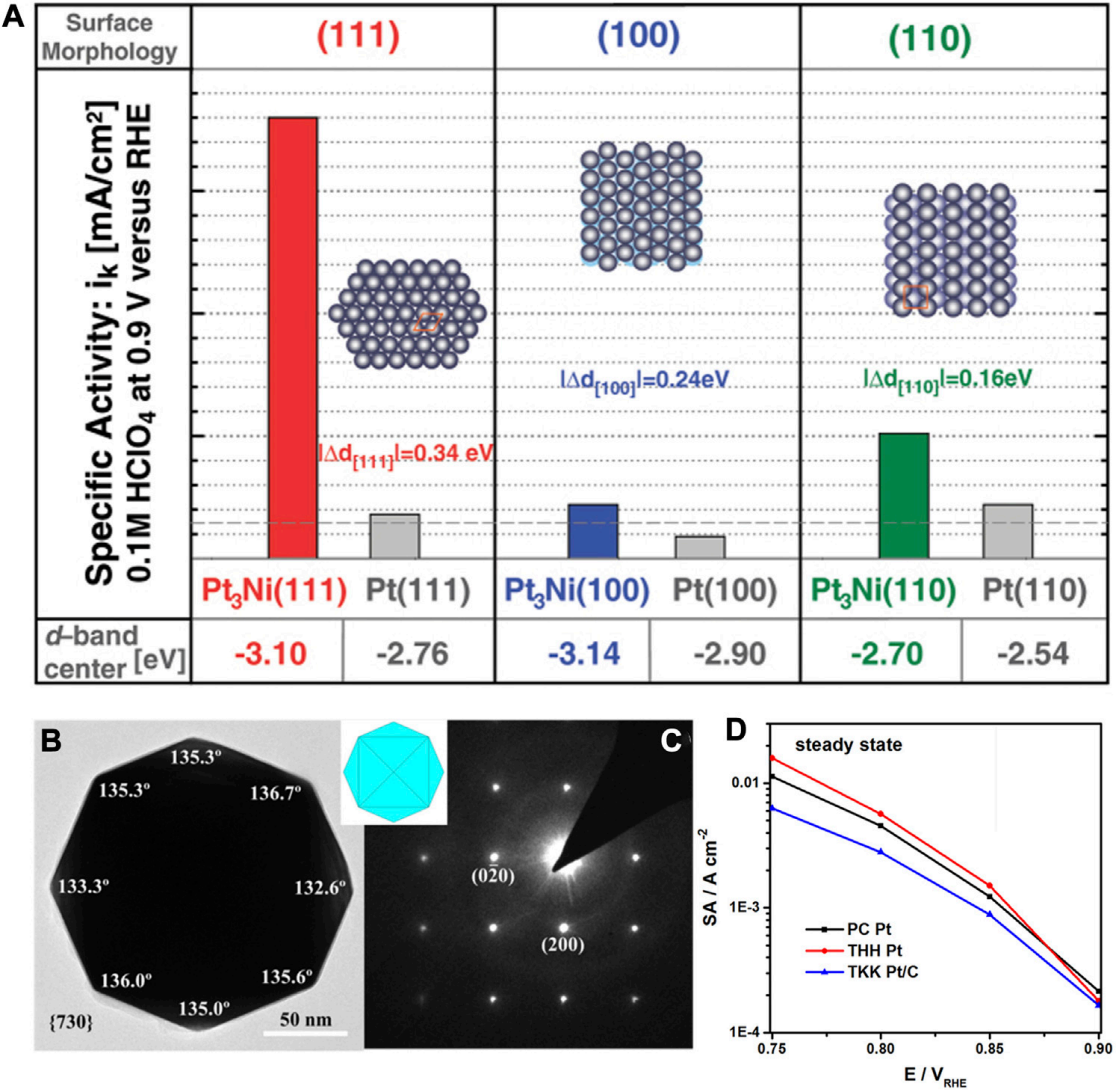
**(A)** Comparison of d-band center and ORR activity for different crystallographic facets of Pt and Pt3Ni alloy. **(B,C)** HRTEM image of Pt nanoparticles with tetrahexahedral (THH) shape. **(D)** Specific activities of polycrystalline Pt, THH Pt and TKK Pt/C in 0.1 M HClO_4_. **(A)** Reproduced with permission (Stamenkovic et al., 2007). Copyright 2007, American Association for the Advancement of Science. **(B–D)** Reproduced with permission (Deng et al., 2017). Copyright 2017, American Chemical Society.

It has long been found that, as shown in Figure 6A, the alloying effect results in the improved Pt_3_Ni activities than the corresponding Pt crystallographic facets. Markovic et al. reported the experimental discovery that the electrocatalytic activity of the (111) facets of single-crystalline Pt_3_Ni alloy for the ORR was 10 times that of Pt (111). Meanwhile, the Pt-skin on different crystallographic surfaces led to a huge difference in the activity for the Pt_3_Ni surface, which generally shows the trend in activity as Pt_3_Ni(100)-skin < Pt_3_Ni(110)-skin <<< Pt_3_Ni(111)-skin (Stamenkovic et al., 2007). More importantly, they found that the Pt_3_Ni(111) surface possessed the decreased d-band center through the density functional theory (DFT), whereby the adsorption energy of related oxygen species decreases, leading to an increased ORR activity. In another work, Sun et al. developed the square-wave potential strategy to synthesize the THH Pt nanoparticles on the electrode, which were enclosed by the high-index crystallographic facets including (730), (210), and (520) (Deng et al., 2017). Under the steady-state conditions, the intrinsic activity of THH Pt nanoparticles was found to be higher than that of polycrystalline Pt as shown in Figure 6D. Furthermore, the THH Pt nanoparticles were stable with a high anti-oxidation feature that can inhibit the transformation in morphology.

Progress has been made in the fundamental electrochemical study of thin-film single-crystalline facets of PGM. A selected summary of synthetic protocols of nanocrystals with low index and high index crystallographic facets can be seen in Table 1. Regulation of the exposed surface of nanocrystals at the atomic scale allows the synthesis of catalysts of highly specific morphology, but challenges remain because they are synthesized mainly in the laboratory instead of being prepared at an industrial scale. Therefore, from the technical point of view, we need to develop a method to synthesize large quantities of catalysts with control over the size and morphology. Besides, the bulk metal single-crystalline facets bear with limited specific surface area and the atomic utilization yield, whereas the nano-catalysts with uniform crystalline facets are usually large in size, leading to limited practical application. Therefore, it is of high necessity to reduce the size of nanocrystals with expected exposed facets. In terms of fundamental research, a deep understanding of the structure-activity relationship at the nanoscale deserves further investigation, which requires the use of ultra-high-resolution characterization techniques and reliable analysis. Furthermore, combining with the machine learning technology, the morphology and exposed crystalline facets of a large number of nanoparticles can be statistically analyzed to assess the structure-activity correlation (Lee et al., 2020). In short, the crystalline facets effect still needs further exploration.

**TABLE 1:** Selected summary of synthetic protocols of nanocrystals with low index and high index crystallographic facets.

Materials	Facets	Shape	Methods	References
Pt	(hk0)	Concave cube	Wet chemistry	Yu et al. (2011)
Pt	(hhl)	TIH	Wet chemistry	Wei et al. (2013)
Pt, Pd, Ru	(hk0)	THH	Dealloying	Huang et al. (2019)
Pd	(hkk)	TPH	Wet chemistry	Zhou et al. (2009)
Pt	(hk0)	TDP	Wet chemistry	Du et al. (2017)
Pt	(hkl)	HOH	Wet chemistry	Xiao et al. (2013)
Pd	(hkl)	DTH	Wet chemistry	Wei et al. (2016)
Pd	(hhl)	TOH	Wet chemistry	Wang et al. (2011)

## Phase Engineering

### Fundamentals

For metals with various crystal phases, the atomic densities of the unit cell, along with the atomic coordination schemes, will affect the morphology and the atomic arrangement at the exposed surface. Meanwhile, the stack mode of metal atoms will also influence the electronic structure (Wang et al., 2019c). Generally, the crystal phases of metal alloy materials can be divided into the intermetallic compound with an ordered atomic arrangement and the random alloy with a disordered atomic arrangement (Bu et al., 2016a). In addition, from the thermodynamic stability point of view, it can also be grouped into the conventional crystal phases and the unconventional phases (Fan et al., 2020). The conventional crystal phases refer to the phases of bulk crystals that show environmental thermodynamic stability. Nanocrystals can sometimes exhibit an abnormal crystal phase, namely the unconventional phase, under specific synthetic conditions (Chen et al., 2020b). For the metallic lattice, all atoms are immobilized in the delocalized electrons, so metal bonds are non-directional, which allows a flexible phase transformation (Chen et al., 2020b).

**FIGURE 7:**
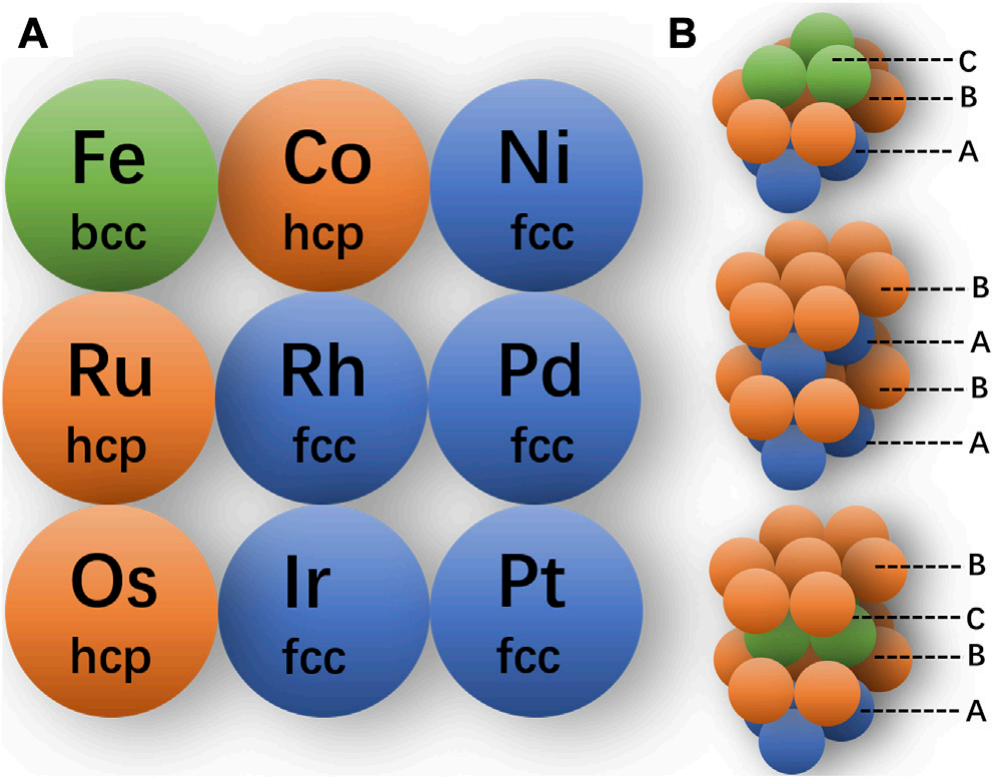
**(A)** Conventional crystal phases of some transition metal. **(B)** Atomic packing models of crystal phase fcc, hcp-2H, and hcp-4H from top to bottom.

For instance, as shown in Figure 7A, for precious metals and some transition metals, their bulk materials tend to form thermodynamically stable phases due to the strong interatomic interactions. In general, the fcc and hcp (hexagonal close packing) structures are the most close-packing structures, with the space occupancy ratio up to 0.74. The Fe adopts a body-centered cubic (bcc) structure, which is non-close-packing with the space occupancy ratio of 0.68. As shown in Figure 7B, the fcc and hcp structures share the same atomic arrangements in the close-packing layer with different stacking orders (Chen et al., 2020b). The fcc and hcp structures can also be called 3C and 2H structures, respectively, wherein the C represents the cubic lattice and H stands for the hexagonal lattice, and the numbers indicate the repeating layers of the lattice. For the hexagonal structure, the stacking order can be modulated along the <100> direction, such as the ABCB mode (as 4H phase) and ABCACB mode (as 6H phase) (Cheng et al., 2018).

### Controlled Synthesis of Unconventional Phases

Most bulk metal materials usually require specific temperature or pressure to induce the phase transformation. For instance, the bcc-fcc phase transformation temperature of the bulk Fe occurs at 1183 K at atmospheric pressure. However, for the nanoscale materials, wherein the surface energy may dominate the total energy, their crystal structure may be different from that of the bulk. Therefore, the phases that may not be stable for the bulk materials can exist in nanomaterials at the same temperature and pressure. For example, the Au generally exist with an fcc structure, but its nanocrystal counterparts with 2H, 4H, and 8H phases have been successfully discovered and synthesized (Fan et al., 2015).

To synthesize nanocrystals with unconventional crystal phases, it is a common strategy to impose special environmental conditions or add special additives. Under high temperature or high pressure, the surface energy of the crystal will be drastically modulated, along with the agitated atomic migration. Hence, the atoms tend to form a new stable phase under such harsh conditions. Another commonly used strategy involves surface modification. Along with the size scaling down to the nanometer, the specific surface area will dramatically increase, and the surface energy will start to play a leading role in the entire system. By covering the surface of NP with the surfactant, the surface energy can be intentionally adjusted.

**FIGURE 8:**
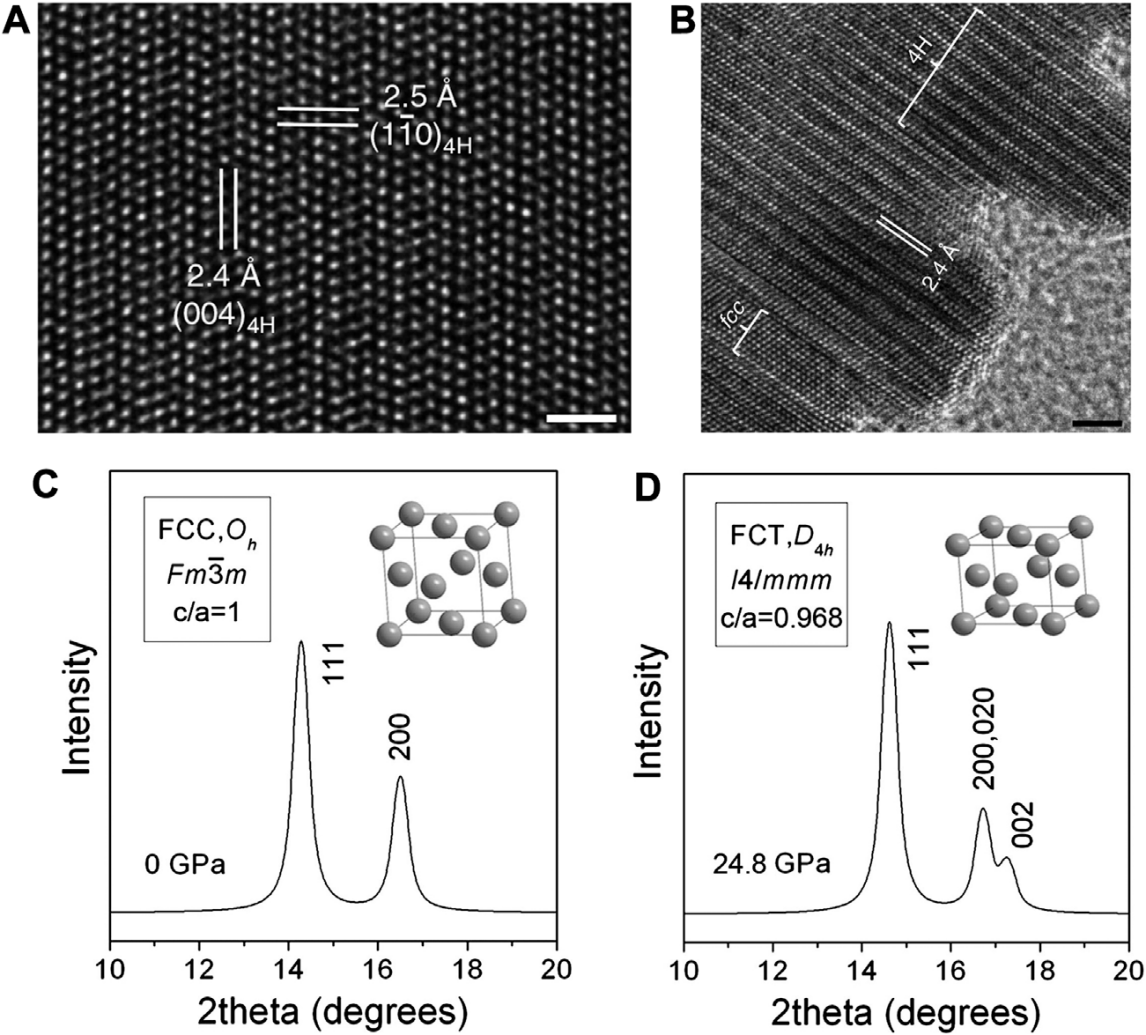
**(A)** HRTEM image of 4H hexagonal Au nanoribbons (scale bar, 1 nm). **(B)** Epitaxial growth of Pd crystals on 4H hexagonal Au nanoribbons (scale bar, 2 nm). **(C,D)** Comparison of XRD patterns of Pd nanocubes before and after compression. **(A,B)** Reproduced with permission (Fan et al., 2015). Copyright 2015, Springer Nature. **(C,D)** Reproduced with permission (Guo et al., 2008). Copyright 2008, American Chemical Society.

Thereby, although the Au, Ag, Pt, and Pd usually adopt the fcc structure, their crystal structures can be appropriately altered by employing new synthetic strategies. Zhang et al. obtained Au nanoribbons by sealing and heating a mixture of HAuCl_4_, oleylamine, hexane, and 1,2-dichloropropane for 16 h, with oleylamine as the surfactant (Fan et al., 2015). As shown in Figure 8A, the lattice structure resolved by the HRTEM agreed well with the simulated 4H hexagonal phase, indicating that the as-synthesized Au nanoribbon had domains of 4H hexagonal phase. Besides, the Pd precursor was further added to form epitaxial Pd NPs on Au nanoribbons, whereby the lattice in the protruding region in Figure 8B dominated by Pd atoms with the same 4H phase as the Au substrate (or a mixed phase of 4H and fcc). In this case, the controlled epitaxial growth is also an effective way to obtain unconventional crystal phases.

Zhang et al. demonstrated the phase modulation of PGM NPs through an epitaxial growth strategy. They synthesized 2H-Pd NPs through the thermal annealing of amorphous Pd NPs in vacuum, based on which the further epitaxial growth of Au NPs on specifically exposed facets of 2H-Pd resulted in various phases (Ge et al., 2020). The (002) crystallographic facet of 2H Pd and the (111) facet of fcc phase Au shared the same close-packing atomic arrangement with the same sixth-fold symmetry which happened to possess almost the same inter-atomic distance. Therefore, the thermodynamically stable fcc Au tends to grow epitaxially on the (002) crystallographic plane of the 2H-Pd seed crystal. For the Au NPs epitaxially grown on the other facets on the 2H-Pd, they tend to display an unconventional 2H phase. What’s more, Xia et al. investigated the phase transformation behavior under high pressures. They loaded Pd nanocubes with an average edge length of about 10 nm in a diamond drill box, implemented the *in-situ* synchrotron X-ray diffraction to monitor structural changes as illustrated in Figures 8C,D, and compressed the sample with high pressure up to 24.8 Gpa (Guo et al., 2008). The sharp (200) and (111) diffraction peaks before applying the pressure confirmed the fcc structure of the Pd nanocubes. However, upon the pressure loading to 24.8 GPa, the (200) peak splits into two peaks with a modified peak intensity ratio of (111)/(200), implying that the Pd nanocube had deviated from the ideal fcc structure and turned into a face center tetragonal (fct, c/a ≠1) structure.

### General Effect of Phase Regulation

**FIGURE 9:**
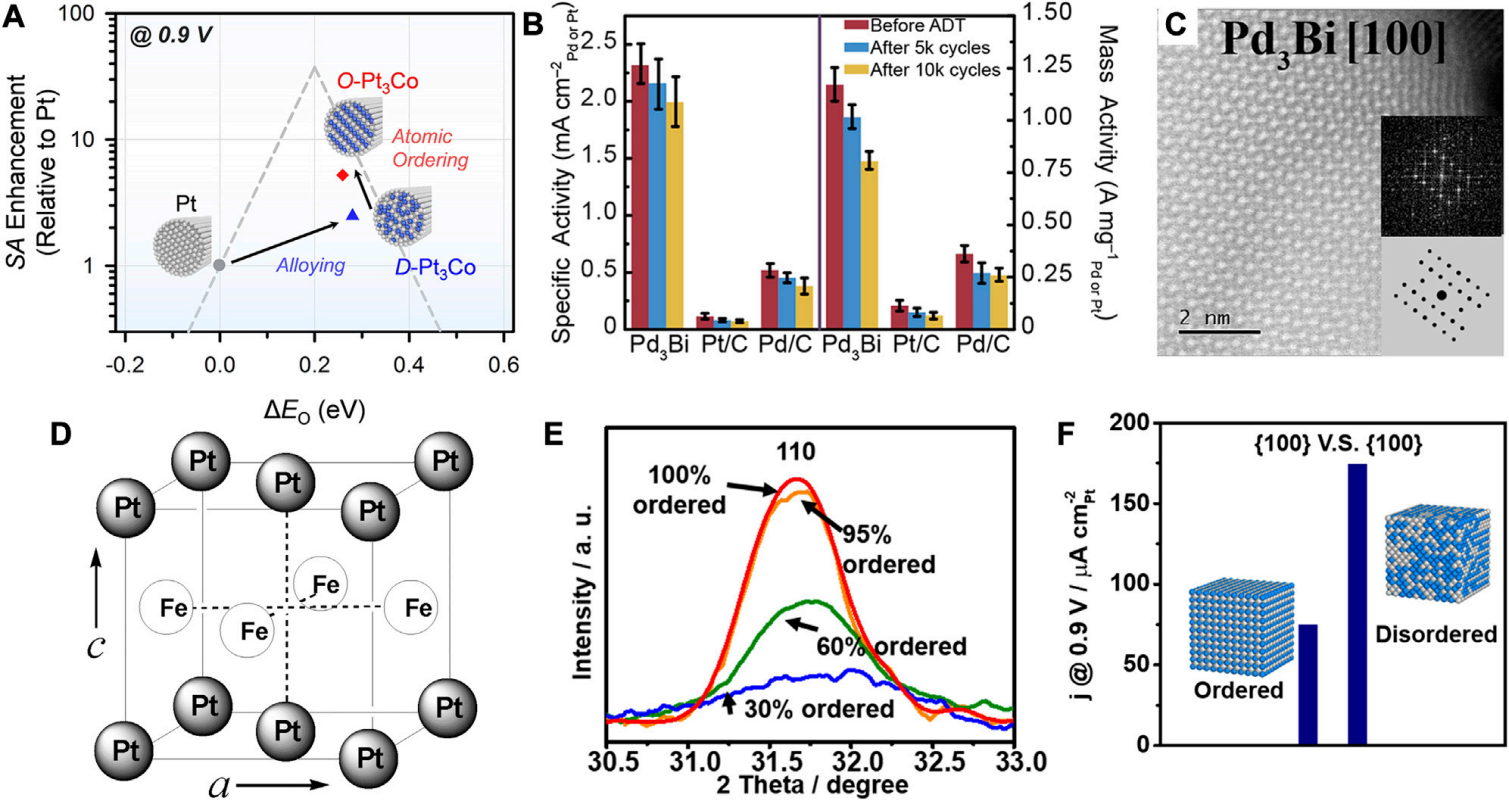
**(A)** Volcanic relationship between ORR activity and O adsorption energy of catalysts. **(B)** Comparison of ORR activity in 0.1 M KOH. **(C)** HRTEM image of Pd_3_Bi (100) crystallographic facet. **(D)** Ordered fct structure of PtFe. **(E)** Comparison of diffraction intensity of (110) diffraction peaks. **(F)** Comparison of Pt_3_Sn ORR activity between 60 and 95% DOs. The illustration is a schematic diagram of ordered and random alloys. **(A–C)** Reproduced with permission (Kim et al., 2019b; Sun et al., 2019).Copyright 2019, American Chemical Society. **(D)** Reproduced with permission (Kim et al., 2010). Copyright 2010, American Chemical Society. **(E,F)** Reproduced with permission (Chen et al., 2020a). Copyright 2020, American Chemical Society.

In addition to the unique structural effect arising from the unconventional crystal phases, the stacking order of metallic atoms, known as ordered and disordered structures, can also greatly influence the catalytic performance. As previously mentioned, the former is referred to as ordered intermetallic compounds and the latter as disordered solid solution alloys. The bonding in the ordered intermetallic alloy shows covalent bonding property and hence results in a lower formation enthalpy and higher thermodynamic stability (Rößner and Armbrüster, 2019). The alloys synthesized through the wet-chemistry generally show disordered atomic arrangement. Intermetallic alloys show a more patterned atomic arrangement and will be more flexible in modulating electronic structures as well as the long-range ordered distribution of active sites. Therefore, the long-range ordering of metal atoms may affect the d-band center relative to the Fermi level by changing the surface electronic density of states (DOS). The catalytic properties of ordered intermetallic nanoalloys are worthy of further systematic exploration. Joo et al. employed thermal reduction of hexachloroplatinic acid and cobalt dichloride to obtain disordered D-Pt_3_Co (disordered-Pt_3_Co) using hollow SiO_2_ as a template (Kim et al., 2019b). Further thermal annealing at 600°C can transform it into ordered O-Pt_3_Co, followed by etching of the silica template to obtain ordered O-Pt_3_Co nanowires. Due to the ordered arrangement of Pt and Co, the bonding within the d orbital was enhanced and the Co imposed a ligand effect on the Pt surface that modulated the chemisorption energy. The adsorption energy of O to the ordered fcc Pt_3_Co was reduced compared to that on the disordered structure, which led to the positive shift of the half-wave potential by 24 mV toward the ORR (Figure 9A).

Generally, thermal annealing at temperatures up to 700°C is of high necessity to promote the atomic diffusion toward an atomically ordered structure. However, the nanoparticles can easily sinter at high temperature, and the carbon from the supporting material may also diffuse into the alloy and influence the catalytic activity. Therefore, their preparation at lower temperature remains a challenge for critical mechanism investigation. By using strong reducing agents or special synthetic systems, such as the microwave and electrochemical techniques, some ordered intermetallic nanoalloys can be directly obtained at milder temperatures. Hall et al. prepared the ordered intermetallic PdBi_2_ by the thermal reduction in oleylamine at 300°C, based on which they further utilized electrochemical dealloying to accomplish the phase transition of ordered intermetallic alloy (Sun et al., 2019). The metallic elements of low melting point exhibits high diffusivity at room temperature, whereas those of high-melting-point show low diffusivity. The Bi, as a low melting point metal, possesses a low vacancy formation energy that enables easier removal of Bi from the surface. As shown in Figures 9B,C, the electrochemical dealloying treatment on PdBi_2_ resulted in the transformation of β-phase PdBi_2_ into an ordered Pd_3_Bi structure through the vacancy diffusion mechanism. The ORR activity of ordered Pd_3_Bi was 14 times higher than that of commercial Pt/C in alkaline, verifying the superiority in the catalytic performance of the ordered intermetallic alloy.

As shown in Figure 9D, Sun et al. synthesized PtFe NPs by the conventional wet-chemistry method (Kim et al., 2010; Zhang et al., 2012). The monodisperse binary PtFe alloy NPs possess a chemically disordered fcc structure, wherein the Fe and Pt atoms are randomly arranged in the lattice reflected from the XRD pattern. To convert the disordered PtFe NPs into the ordered structure, they employed a thermal treatment protocol with 5 at% H_2_ in Ar atmosphere at 750°C for 6 h, before which the MgO was deposited on the PtFe surface to prevent the sintering of NPs at high temperature. After annealing treatment, the emergence of (001) and (110) characteristic diffraction peaks indicated that the disordered PtFe had transformed into an ordered fct structure. The Fe and Pt atoms were arranged in an ordered pattern, with alternating atomic layers in the fct structure. As a novel design, the MgO coating can be further removed by acidic etching. The chemical stability of the PtFe NPs with disordered fcc structure and ordered fct structure was evaluated by measuring the amount of leached metals in 0.5 M sulfuric acid. It was found that the dissolution of Fe from the fct PtFe was almost negligible, whereas a huge amount of Fe was leached from the fcc PtFe. They proposed that the structure transformation resulted in a dramatic change in magnetic response from super-paramagnetism to ferromagnetism, which influenced the chemical stability. Besides, compared with fcc PtFe, the ORR reaction kinetics of the fct PtFe was found to be more efficient in 0.5 M H_2_SO_4_.

For binary alloys, ordered and disordered structures represent two basic crystalline structures. In most cases, the orderly intermetallic compounds will increase the catalytic activity due to the enhanced atomic coupling effect. But the trend may not always stand true depending on the specific variety and morphology of the alloy. In some cases, the high regularity of the binary alloy may also show negative effects.

Besides, Tilley et al. synthesized Pt_3_Sn nanocubes with different degrees of alloy ordering, e.g., 95, 60, and 30%, with the same size and composition (Chen et al., 2020a). As shown in Figures 9E,F, the degrees of ordering (DO) were calculated based on the integrated intensity of the characteristic (100) diffraction peak. The low segregation energy of Sn atoms in Pt_3_Sn alloy ensured their easier migration than Pt atoms during the annealing. The EXAFS result showed that the nearest coordination number of Sn changed greatly with the DO, indicating that the migration of Sn could lead to different DOs. The EXAFS and XPS measurements confirmed that Pt-Pt and Sn-Pt bond distances, element distribution, and valence were not changed. Taken together, it was indicated that the ORR activities were closely related to the DOs. The Pt_3_Sn with 60% DO exhibited a 2.3-time activity compared with that with 95% DO, which may be induced by disordering on the surface of nanocubes. The decreasing DO led to easier dissolution of Sn atoms and created concave defects on the surface during the activation, which was commonly considered as highly active sites (Calle-Vallejo et al., 2015).

**FIGURE 10:**
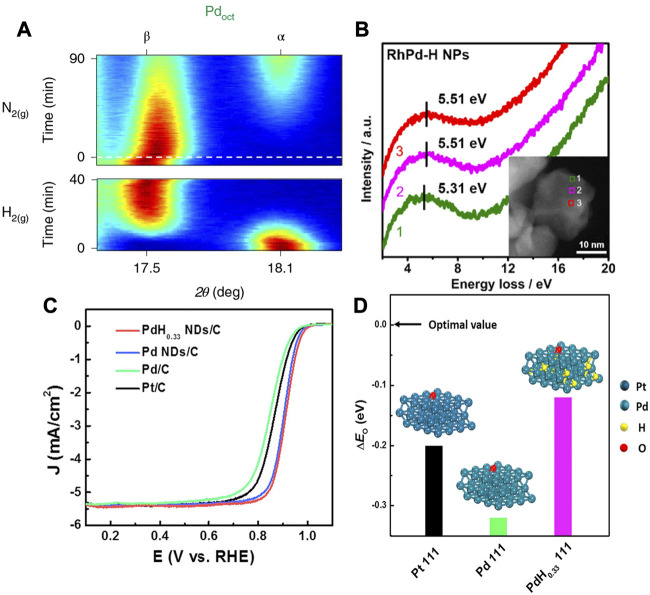
**(A)** *In situ* XRD shows the transition between the α phase (N_2_ atmosphere) and β phase (H_2_ atmosphere) for PdH_x_ octahedrons (Oct). The red represents high intensity, blue represents low intensity. **(B)** Electron energy loss spectra of RhPd-H nanoparticles at different positions. It verifies the existence of interstitial H atoms in the RhPd-H lattice. **(C)** Linear sweep voltammetry curve comparison of ORR activity of PtC, PdC Pd NDs/C and PdH_0.33_ NDs/C in 0.1 M KOH electrolyte. **(D)** The surface O adsorption energy calculated by DFT based on the Pt (111), Pd (111) and PdH_0.33_ (111) models. **(A)** Reproduced with permission (Johnson et al., 2019). Copyrights 2019. Springer Nature. **(B)** Reproduced with permission (Fan et al., 2019). Copyrights 2019. American Chemical Society. **(C,D)** Reproduced with permission (Wang et al., 2019b). Copyrights 2019. Elsevier.

The Pd is considered as a special PGM element in terms of its strong absorption of hydrogen. The hydrogen species such as H_2_ or H^+^, can adsorb on the metal surface and subsequently absorb into the metal lattice and form the palladium hydride (PdH_x_) forming the two unique fcc phases under specific conditions (Johnson et al., 2019). The α phase is formed with low hydrogen content (x < 0.01), which can be further transformed into a lattice-expanded β phase at a high hydrogen concentration (x ≈ 0.7) (Narayan et al., 2016; Sherbo et al., 2018). The two phases could coexist for x in the range 0.01∼0.7. The distribution of H in the PdH_x_ depends on the morphology and size of NCs, which can be detected by the neutron scattering technique (Baldi et al., 2014). For a typical PdH single crystal, each Pd atom is surrounded by eight H atoms with an octahedral arrangement (Narayan et al., 2016). As shown in Figure 10A, the octahedral Pd NCs may undergo an α-to-β phase transition in the H_2_ atmosphere, and transforms back to α phase as the H_2_ is removed (Johnson et al., 2019).

The electrocatalysis of the Pd-based catalysts was further explored to investigate the structural influence of α and β phases. As shown in Figure 10B, Cui et al. reported that the interstitial hydrogen atom in RhPd can effectively boost the HER activity by adjusting the electronic structure (Fan et al., 2019). The peaks in electron energy loss spectrum (EELS) corresponded to the plasmon resonance for the RhPd-H, verifying the transfer of electrons from metal atoms to H atoms due to the modulation of interstitial H (Fan et al., 2019). In another work, Song et al. synthesized β-phase PdH_0.33_ dendrites with exposed (111) crystallographic facets (Wang et al., 2019b). The O adsorption energy ΔE_0_ can be used to assess the ORR activity as a descriptor. The higher ΔE_0_ is not favorable for the desorption of O and OH. As shown in Figures 10C,D, the DFT showed that the β-phase PdH_0.33_ dendrites displayed an oxygen adsorption energy close to optimal value compared with the Pt (111) and Pd (111). The specific activity and mass activity of β-phase PdH_0.33_ dendrites were found to be 5.2- and 5.7-time higher than that of commercial Pt/C, respectively.

The unconventional crystal phase with abnormal atomic arrangement has been successfully synthesized and demonstrated with excellent electrocatalytic activities (Table 2). However, challenges remain in various aspects. From the synthetic point of view, the synthesis of NCs with unconventional crystal phases remains difficult. For instance, their synthetic yields are usually low, with difficulty in simultaneous control over the phase and the morphology. The mechanism investigation into the formation of irregular crystalline phases is usually difficult. Concerning stability, although unconventional crystal phases could possess novel properties, they are usually less stable, which limits their application. For binary or ternary alloys, the intermetallic alloys usually display promising electrocatalytic performances especially in terms of stability. However, the transformation of random alloys to ordered alloys usually requires annealing treatment with high temperature, which may lead to the ripening of NPs and the formation of carbon-coated shells. So far, the typical atomic ratio of PGM in the intermetallic compounds is 75% or 50%. It is necessary to further reduce the PGM element to lower the cost (Rößner and Armbrüster, 2019). For the Pd-based alloy, although it can easily transform into the metallic hydride phase, the H in the hydride may gradually be released in the catalytic environment, which may devastate their durability of performance. Furthermore, at the macro-micro scale, the idea of phase engineering could be extended to the design of self-assembled superlattice structure, such as a Pt-Pd binary superlattice material assembled by two types of NPs (Kang et al., 2013).

**TABLE 2:** Summary of methods for synthesizing new crystal structures of PGM based NCs.

Materials	Crystal structure	Synthetic method	References
**PdM (M = Zn, Cd, CdZn)**	Ordered fct nanosheets	High temperature wet chemical reduction	Yun et al. (2019)
**AuPd**	4H hcp phase	Epitaxial growth in Au substrate	Fan et al. (2015)
**Pd**	fct nanocube	High pressure	Guo et al. (2008)
**PtFe**	Ordered fct NPs	High temperature annealing	Kim et al. (2010)
**PtFeAu**	Ordered fct NPs	High temperature annealing	Zhang et al. (2012)
**Pd**	bct film	Epitaxial growth in W (001) substrate	Jona and Marcus (2002)
**Pt**_**3**_ **Co**	Ordered fcc NWs	High temperature annealing	Kim et al. (2019b)
**Pd**_**3**_ **Bi**	Ordered fcc NPs	Wet reduction + electrochemical dealloying	Sun et al. (2019)
**Pd**_**3**_ **Pb**	Ordered fcc tripod	Wet chemical reduction	Bu et al. (2018a)
**Pt**_**3**_ **Sn**	Ordered fcc nanocube	Wet chemical reduction	Chen et al. (2020a)
**Pd**_**3**_ **Pb**	Ordered fcc nanosheet	Wet chemical reduction	Bu et al. (2018b)
**PtBi**_**2**_	Ordered fcc NWs	Microfluidic reactor	Zhang et al. (2015)
**Pt**_**3**_ **Co**	Ordered fcc NPs	High temperature annealing	Wang et al. (2013)
**PdCoAu**	Ordered fcc NPs	High temperature annealing	Kuttiyiel et al. (2014)
**PdCu**	Bcc	Wet chemical reduction	Park et al. (2020)
**β-PdH**	Fcc	Wet chemical reduction	Zhao et al. (2015)

M, metal element.

## Strain Engineering

### Fundamentals of Lattice Strain

Strain can effectively influence the electronic structures of metals, thereby affecting the adsorption energy of oxygen molecules, which can be employed as a powerful strategy to regulate the electrocatalytic activity of alloys. The lattice strain can usually be characterized by the shift of the diffraction peak on the XRD patterns. As the catalytic reactions occur mainly on the surface, most studies focused on the strain within the surface, which can be characterized by XRD or HRTEM (Li et al., 2018; Tang et al., 2019). In this regard, the ubiquitous in-plane uniaxial and biaxial strain are found to influence the catalytic process (Bu et al., 2016b; Li et al., 2020c). More recently, it is considered that the strain perpendicular to the basal plane may also be of high importance (Wu et al., 2020). The above strains are all grouped as macro-strains, distinguished from the so-called micro-strain that is thought to be related to the defects which will be discussed in *Structural Defect Engineering*. The micro-strain, induced mainly by lattice distortion, can usually be recognized by the broadening of the diffraction peak on the XRD patterns. Based on the d-band model, the position of the d-band center plays a decisive role in the adsorption energy and activation energy barrier. For the Pt, an average of merely 1% lattice strain can deviate the 5d band center by 0.1 eV, which can significantly influence the adsorption/desorption of the reaction intermediate on the surface (Bu et al., 2016b; Luo and Guo, 2017; Xia and Guo, 2019).

The current strategies for the regulation of the strain mainly include the following methodologies:

(1) By appropriately employing the lattice mismatch of different metals, the strain effect can be demonstrated by precise construction of the core@shell structure through the bottom-up epitaxial growth, or top-down chemical and electrochemical removal of surface atoms. The epitaxial interface ensures lattice continuity and hence the difference in lattice parameters between the core and shell layers will induce a lattice strain that can influence a few atomic layers at the interface (Bu et al., 2016b; Li et al., 2018; Li et al., 2020a; Liu and Zhang, 2020).
(2) The metal catalysts can be deposited on substrate materials to generate the intrinsic strain through the strong physical or chemical interaction at the interface. Besides, the lattice expansion or compression of the substrate, induced by the external force or temperature variation, can also be transferred to the metal catalyst and induce the strain in the metal (Du et al., 2015; Wang et al., 2016).
(3) For ultra-thin two-dimensional materials, the in-plane intrinsic strain can be generated by anisotropic atomic interaction or geometric deformation (Wang et al., 2019a; Luo et al., 2019).

### Strain in core@shell Structure

**FIGURE 11:**
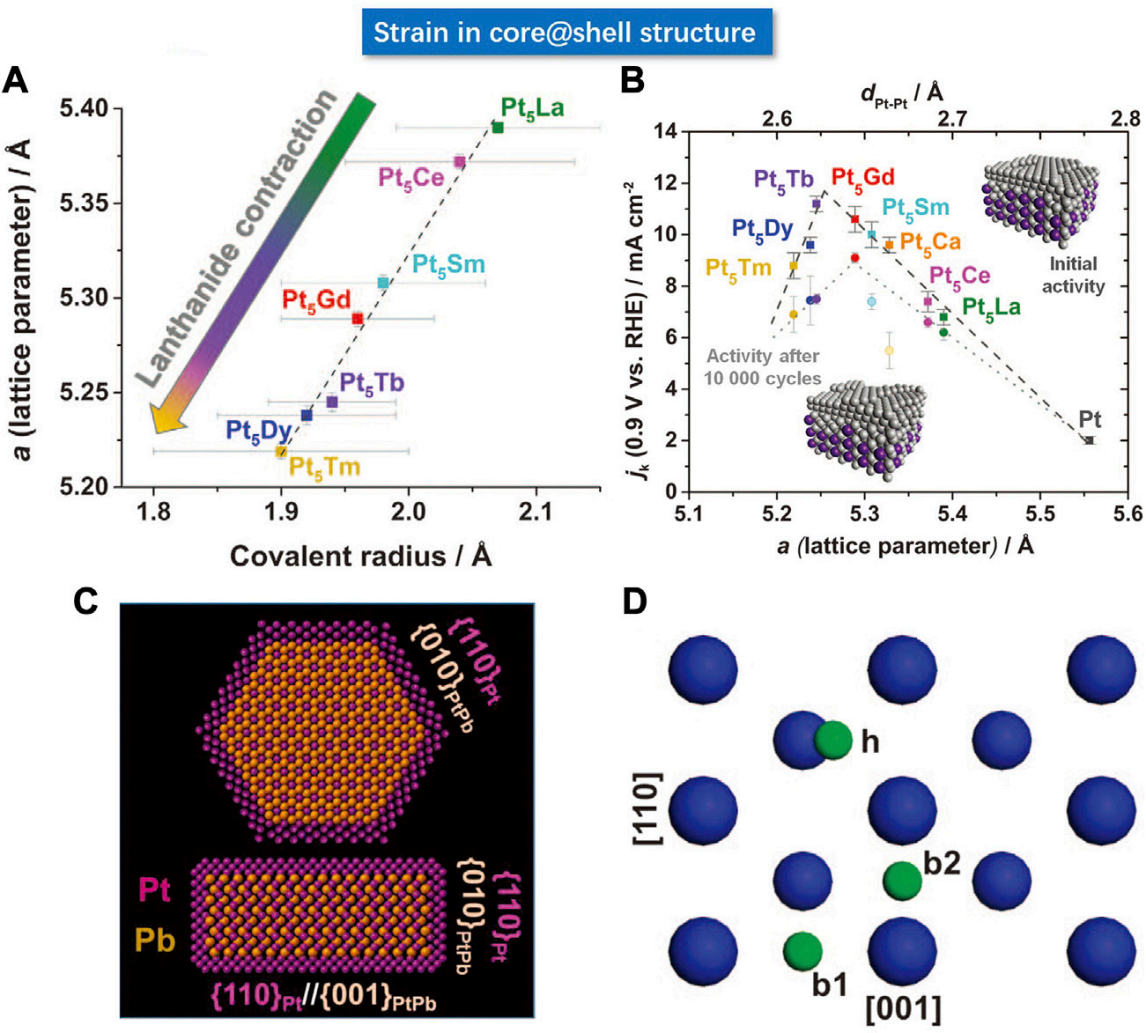
**(A)** Relationship between lattice parameters and covalent radius of lanthanide (La, Ce, Sm, Gd, Tb, Dy, Tm, etc.), wherein the scattered points are approximately linear. **(B)** Volcanic relationship between lattice parameters and ORR kinetic current. **(C)** Atomic model of PtPb@Pt hexagonal nanoplates. **(D)** Schematic of (100) crystallographic facet, blue balls represent Pt atoms. **(A–D)** Reproduced with permission (Bu et al., 2016b; Escudero-Escribano et al., 2016). Copyright 2016, American Association for the Advancement of Science.

Chorkendorff et al. reported a series of Pt-based alloy consisting of the lanthanide metal elements, including the La, Ce, Sm, Gd, Tb, Dy, Tm, etc. They leached the surface lanthanide atoms to form a Pt shell on the surface, whereby the surface Pt-Pt distance would change due to the lattice mismatch between the Pt shell and the PtM core (Escudero-Escribano et al., 2016). As summarized in Figure 11A, the Pt-Pt bond length has a strong dependence on the type of M element in the core region. As shown in Figure 11B, the lattice parameters of the as-prepared core-shell structures are linearly related to the covalent atomic radius of lanthanides by XRD. More specifically, the Tm has a radius lower than La, hence the lattice parameter of Pt_5_Tm is lower than Pt_5_La, which is verified by the calculated Pt-Pt bond length from the XRD results. The intrinsic ORR current density is found to have a volcano-shaped dependence on the Pt-Pt bond length.

For the Pt (111) crystallographic facet, it has been commonly accepted that the compressive strain can weaken the adsorption of O species, whereas the tensile strain increases the adsorption energy for O. Based on the previous discussion on the intrinsic strong adsorption to oxygen of the Pt surface, it means that the tensile strain is not favorable to the ORR reaction. Huang et al. developed a core-shell system with enhanced ORR performance induced by tensile strain. They synthesized hexagonal PtPb@Pt nanoplates (Bu et al., 2016b). The Pt shell consists of 4–6 atomic layers with the (110) crystallographic plane exposed on the basal facets and the edges of the nanoplates. Since the Pb atoms are larger than Pt atoms, the tensile strain would present in the nanoplate. The author investigated the PtPb (010) and PtPb (001) crystal planes. They found that compared with pure Pt, tensile strain is generated in one axis, while the compressive strain is generated in the other axis. As shown in Figure 11C, the biaxial strain of the core layer will induce in-plane biaxial strain on the Pt (110) crystal plane. As shown in Figure 11D, the blue and green spheres represent the Pt and O atoms, respectively. The larger blue spheres represent the Pt atoms at the topmost layer, and the smaller blue spheres represent the Pt atoms at the sub-surface layer. They calculated the O adsorption energy at three adsorption sites on the Pt (110) surface with the DFT and found that the oxygen adsorption energy depended strongly on the biaxial strain. The results verify that the oxygen adsorption energy for ORR under the biaxial strain is more favored on the hollow (h) sites than on the Pt (111).

### Strain in Heterojunction Interface

**FIGURE 12:**
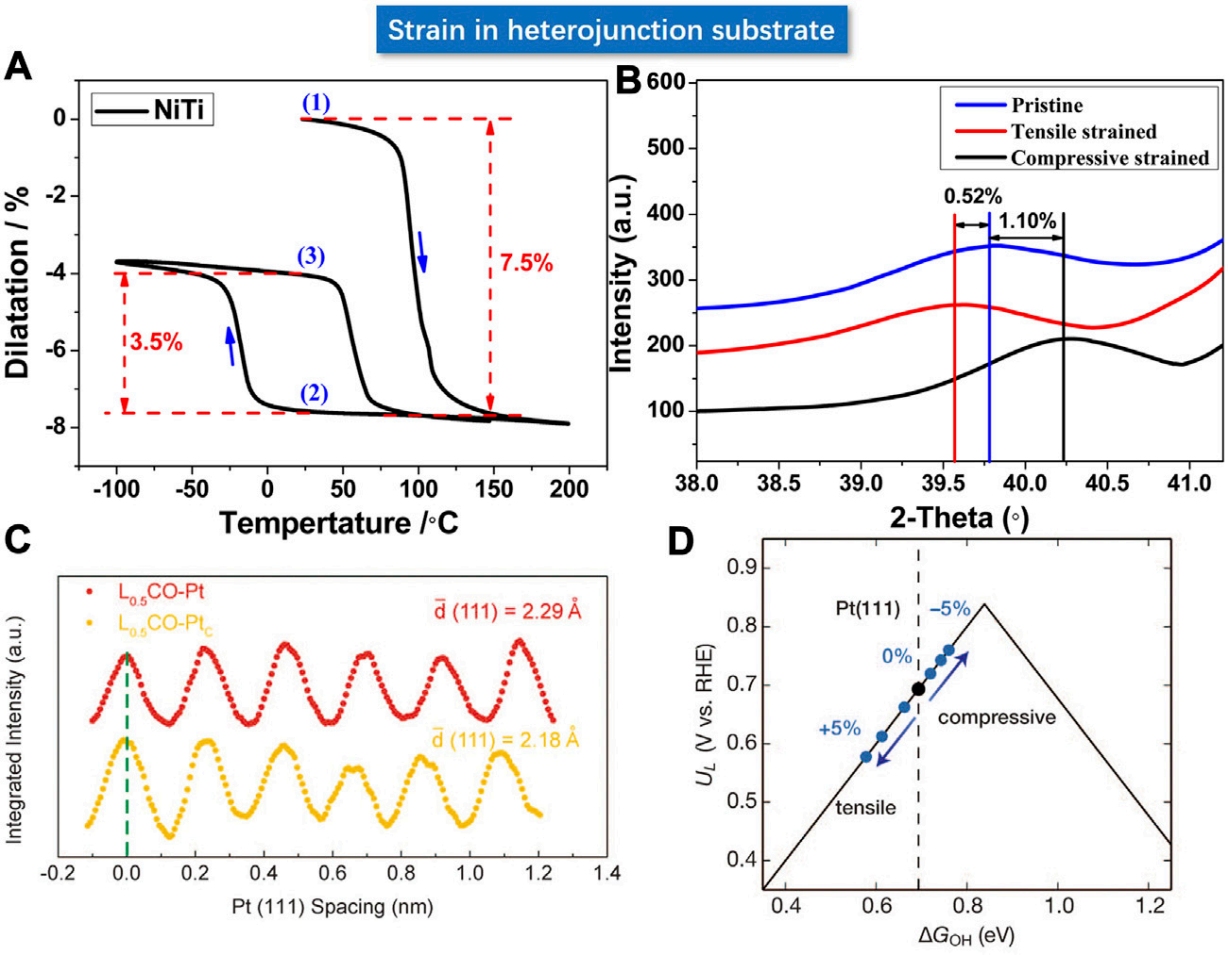
**(A)** Deformation of shape memory alloy NiTi under different thermal cycling treatments. **(B)** Variation of strain on Pt film. **(C)** Lattice spacing of Pt (111) crystallographic facet under charge (red) and discharge (yellow) respectively. **(D)** Volcanic relationship between activity and strain based on the experimental result. **(A,B)** Reproduced with permission (Du et al., 2015). Copyright 2015, American Chemical Society. **(C,D)** Reproduced with permission (Wang et al., 2016). Copyright 2016, American Association for the Advancement of Science.

The regulation of strain in the target material is considered as a challenge due to the difficulty in precisely loading the strain. Some novel strategies have been reported using a substrate material with controllable lattice parameters to load the strain. Bard et al. deposited Pt nano-polycrystalline films of 5 and 10 nm thick on a NiTi substrate at room temperature (Du et al., 2015). The NiTi alloy could operate as a typical shape memory alloy via a unique phase transformation mechanism (as shown in Figures 12A,B), enabling a flexible and precise strain engineering regulation over the deposited Pt film with desirable lattice expansion and shrinkage. The XRD verified that a 7.5% shrinkage of the NiTi substrate can only lead to an average of 1.10% compressive strain in the 10 nm Pt film, and a 3.5% expansion of NiTi substrate led to 0.52% tensile strain in Pt, wherein the “discounted” loading of strain may be induced by the relief of stress at the interface. Consequently, the interfacial Pt layer should be gifted with the maximum elastic strain, whereas increasing film thickness could decrease the strain on the surface Pt layer and hence attenuate its effect on activity. For the 5 nm Pt film, the kinetic rate constant upon the loading of compressive strain increased by 52%, along with a positive shift of 27 mV for the half-wave potential. In contrast, the tensile strain results in a 35% decline in the rate constant and a 26 mV negative shift for the half-wave potential, which echoed with the d-band theory. In addition, shape memory polymers can also be considered as substrate materials (Xia et al., 2020).

Inspired by the lithium intercalation mechanism in lithium-based batteries, Cui et al. used the LiCoO_2_ as catalyst support and fabricated Pt NPs on the support with strong interaction (Wang et al., 2016). The LiCoO_2_, a typical positive electrode material used in lithium-ion batteries, is endowed with precisely controllable expansion or contraction in volume and lattice parameters in the electrochemical charging and discharging processes. As shown in Figures 12C,D, the LiCoO_2_ displayed about 5% lattice compression and tensile strain through the lithium intercalation operation. The volume change induced the lattice strain in Pt nanoparticles through the strong interfacial interaction. The catalytic activity of Pt NPs in alkaline solution could hence be modulated in a flexible manner. With precise control in between the compressive strain and tensile strain, the activity can be continuously and significantly modulated by +90% (enhancement) and −40% (decline). The experimental results were in good agreement with the predictions of DFT calculations that predicted the weakened binding energy of OH* with uniaxial compression strain.

### Intrinsic Strain in Two-Dimensional Nanostructure

**FIGURE 13:**
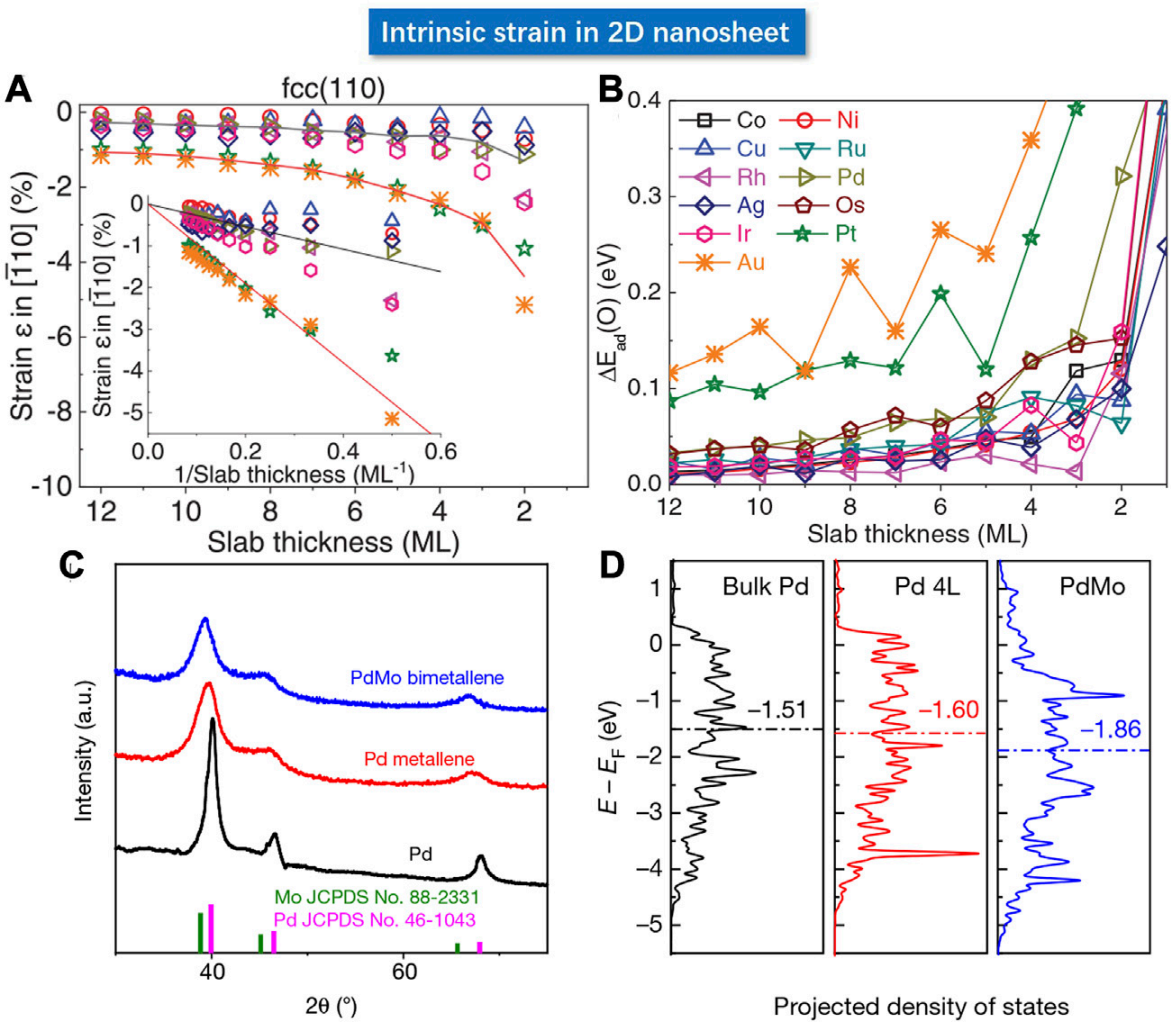
**(A)** The relationship between the strain and thickness of coin metal nanosheets by calculation. The inset shows that the magnitude of strain is inversely proportional to the thickness. **(B)** Relationship between nanosheet thickness and O adsorption energy by DFT calculation. **(C)** Powder XRD patterns of PdMo bimetallene, Pd metallene and Pd. **(D)** Calculated d-band center of bulk Pd, Pd nanosheet (4L) and PdMo bimetallene. **(A,B)** Reproduced with permission (Wang et al., 2019a). Copyright 2019, American Association for the Advancement of Science. **(C,D)** Reproduced with permission (Luo et al., 2019). Copyright 2019, Springer Nature.

In addition to the interface-based strain engineering strategies, the strain can also be generated through geometric deformation or surface atomic reconstruction. Cracking the bulk material to create new surfaces usually results in a redistribution of electrons in the metal, which can induce the collective inter-atomic force for the surface atoms that tends to reduce the surface energy (Wang et al., 2019a). Therefore, when the bulk atoms turn into surface atoms due to the structural changes, there will be changes in the inter-atomic distances. For instance, for the surface atoms in the two-dimensional ultrathin films, the in-plane inter-atomic interactions are similar to that in the bulk material, whereas the inter-plane atomic interactions will be dramatically different induced by the asymmetric coordination, leading to an in-plane strain for the 2D materials. A first principle calculation for the Pt-group metals by Wang et al. revealed that these metals possessed an inherent surface strain due to the inter-atomic force that can achieve a surface stress up to 105 atm (Wang et al., 2019a). The surface stress may show very little effect on the bulk material, but it can impose a significant effect on the lattice of the 2D ultra-thin film and induce an in-plane compressive strain and a change in the inter-atomic-layer spacing, which explains the intrinsic strain in ultrathin two-dimensional materials. As shown in Figure 13A, the DFT result showed that the intrinsic strain can reach 10% for the monoatomic layer 2D materials. Generally, the in-plane intrinsic strain is inversely proportional to the thickness. Therefore, regulation of thickness can be employed as an effective strategy to modulate the lateral intrinsic strain. As shown in Figure 13B, the slab strain induced the change in the electronic structure and hence affected the adsorption energy of the oxygen intermediate. The theoretical correlation between the adsorption energy and the ORR overpotential revealed a volcano-shaped dependence. The Pd nanosheet with 3 atomic layers showed a significantly enhanced ORR activity from the rotating disk electrode (RDE) test.

Strain can also be generated in 2D material through the geometric effect. Guo et al. used a one-pot method to synthesize highly curved ultra-thin Pd metallene and PdMo bimetallene (Luo et al., 2019). The atomic force microscope (AFM) determined a thickness of 0.88 nm with only several atomic layers. Due to the highly curved geometry, the Pd metallene displayed a lattice expansion compared to that in the bulk Pd, whereas the PdMo bimetallene showed a 1.4% tensile strain as shown in Figure 13. The DFT calculations suggested a linear relationship between the oxygen adsorption energy of PdMo bimetallene and the strain, wherein the positive and negative values represent the tensile and compressive strain, respectively. The red dotted line signifies the optimal ΔE_0_ value at 1% tensile strain consistent with the experimental facts. Meanwhile, the d-band electron density of the surface was calculated, wherein the horizontal dashed line indicates the lowered d-band center in PdMo bimetallene. According to the experimental results, the ORR mass activity of the optimized PdMo bimetallene reached 16.4 A/mg_Pd_ (0.9 V), as the champion activity of the Pd-based catalyst reported so far.

It is generally believed that the decisive factor of ORR lies in the binding/adsorption energy of the reaction intermediate to the catalyst surface, which eventually depends on the electronic structure on the surface. In recent years, theoretical studies have shown that the down-shift of the center of the d-band can weaken the binding energy (Table 3). Strain engineering modulates the catalytic activity by changing the inter-atomic distance. From the geometric point of view, smaller inter-atomic distance is favored by the ORR kinetics.1 In this respect, the electronic structure and catalytic activity can be achieved by adjusting the surface strain. Active strain and passive strain could be classified according to the source of the strain in nanocrystals. The active strain is mainly generated in the ultrathin nanosheet, whereas the passive strain is generated from the lattice mismatch (Escudero-Escribano et al., 2016).

**TABLE 3:** Summarized examples employing strain engineering for ORR studies.

Catalyst	Electrolyte	SA (mA/cm2Pt + Pd)	MA (A/mgPt + Pd)	ECSA (m2/gPt + Pd)	References
**Au NWs @Pd@ PEI**	0.1 M KOH	0.4	0.29	72.5	Cleve et al. (2017)
**PtFe@Pt**	0.1 M HClO_4_	—	0.7	—	Li et al. (2018)
**Pd**_**2**_ **FeCo@Pt**	0.1 M KOH	—	5.5	—	Xiao et al. (2017)
**Pd**_**2**_ **FeCo@Pt**	0.1 M HClO_4_	—	2.1	—	Xiao et al. (2017)
**Pd**_**3**_ **Pb@Pd nanosheet**	0.1 M KOH	1.5	0.6	40	Tang et al. (2019)
**Pd@PdFe Icosahedron**	0.1 M KOH	1.2	0.3	25	Li et al. (2020c)
**PtY**	0.1 M HClO_4_	—	3.05	—	Hernandez-Fernandez et al. (2014)
**PdMo bimetallene**	0.1 M KOH	11.5	16.5	143	Luo et al. (2019)
**PdMo bimetallene**	0.1 M HClO_4_	0.48	0.65	135	Luo et al. (2019)
**PtPb@Pt nanoplate**	0.1 M HClO_4_	7.8	4.3	55	Bu et al. (2016b)
**Pd nanosheet**	0.1 M KOH	10.42	6.36	61	Wang et al. (2019a)
**Pd nanosheet**	0.1 M HClO_4_	0.61	0.35	58	Wang et al. (2019a)

SA, specific activity; MA, mass activity; ECSA, Electrochemical Surface Areas.

However, some challenges remain for future development of strain engineering. Theoretically, the passive strain exists mainly at the interface, which can be influential across up to 4–5 atomic layers (Liu and Zhang, 2020). The strain could further relax during the continuous operation or cycling when destructing the interface, along with the convergence in composition. Therefore, a more stable interface needs to be constructed to sustain the strain. In addition, the strain is generally characterized by the shift of XRD diffraction peaks. However, the XRD results only reflect the average strain of the entire powder sample. Therefore, a more precise three-dimensional strain characterization method is required. Besides, for the strain in the alloyed NPs, due to the influence of other factors, it is impossible to accurately distinguish the dominant factor between the strain effect and the ligand effect.

## Structural Defect Engineering

### Fundamentals of Structural Defect

**FIGURE 14:**
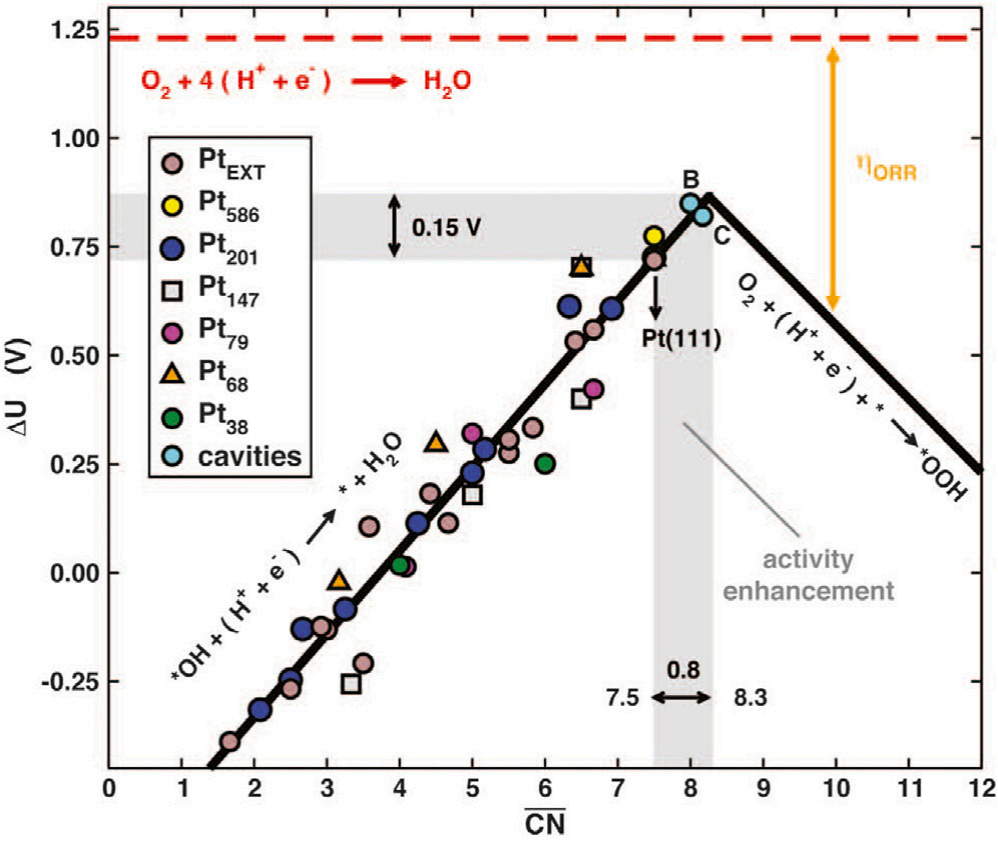
Coordination-activity plot diagram. Potentials for the two limiting steps on extended surfaces and NPs. Reproduced with permission (Calle-Vallejo et al., 2015). Copyright 2015, American Association for the Advancement of Science.

Crystal defects represent the positions wherein the periodic arrangement in the crystal structure are destroyed, which will cause the redistribution of the chemical coordination and electronic structure at the surface. From the size or dimension point of view, the crystal defect can be divided into low-dimensional point defects, line defects, and high-dimensional plane defects, body defects (Jia et al., 2019). For instance, common low-dimensional defects in PGM catalysts include vacancy, dislocation, and grain boundary, etc. The high-dimensional defects, namely the body defects such as the cavities, consist mainly of local vacancies, dislocations, grain boundaries, and collective low-dimensional micro-defects on the surface. Therefore, the body defects can be seen as a mixture of micro-defects (Jia et al., 2019; Li et al., 2020b). In terms of surface geometry, the creation of these surface defects may lead to the local surface roughening, whereby the ceased periodic atomic arrangement generates the local lattice distortion and hence the enrichment of surface micro-strains. Therefore, the micro-strain induced by the micro-defects may lead to the varied Pt-Pt bond length and the modified local electronic structure. As for a continuously changing electronic structure, there will be a much higher possibility that a particularly suitable active center could exist at the defected region with the optimal adsorption energy to the oxygen species. In this way, the adsorption of oxygen-related intermediates can be optimized to regulate the intrinsic activity. From the chemistry point of view, the “roughening” of surface atoms induces irregular atomic arrangement and hence the irregular coordination state. Especially, the coordination number of atoms at the edges and vacancies will be reduced, such as the concave catalytic sites (Calle-Vallejo et al., 2015). The generalized coordination number (GCN) is a descriptor of local structure based on geometric principles. By calculating the coordination number of the neighboring atoms, one may predict the optimized geometry of the best catalytic site. In many cases, the coordination number agrees well with the energy descriptor. Federico Calle-Vallejo et al., exemplifying with Pt, calculated the GCN values of the classic Pt (111) crystallographic facet to be 7.5 (Calle-Vallejo et al., 2015). As shown in Figure 14, according to the coordination-activity volcano diagram, the GCN value of the best catalyst should reach 8.3. The result showed that introducing low-dimensional defects on the ideal crystallographic facet could change the local coordination number, thereby enhancing the activity of a single active site. They used different methods to fabricate vacancy defects on the Pt (111) crystal facets. As a result, the activity of the catalysts increased by 3.5 times in their experiment (Calle-Vallejo et al., 2015). With a low coordination number, the removal of the *OH in the elementary reaction plays a decisive role, which dominates the left branch of the volcano plot in the coordination-activity diagram, whereas a high coordination number may arise the decisive role of the formation of *OOH that dominates the right branch (Calle-Vallejo et al., 2015). Therefore, defects are considered as a necessity to achieve the appropriate coordination number for the perfect crystallographic facets. However, due to the high surface energy of the defects, a compromise has to be taken between the activity and stability. Vlachos et al. used the Pareto optimal allocation method to calculate the ORR activity of Pt that can stably retain defects after quenching. By considering both the surface energy and coordination number, they proposed that the density of highly active sites was higher when formed on the defective surface rather than the perfect crystallographic facet, which served as a guide for the defect engineering (Núñez et al., 2019).

### General Effect

**FIGURE 15:**
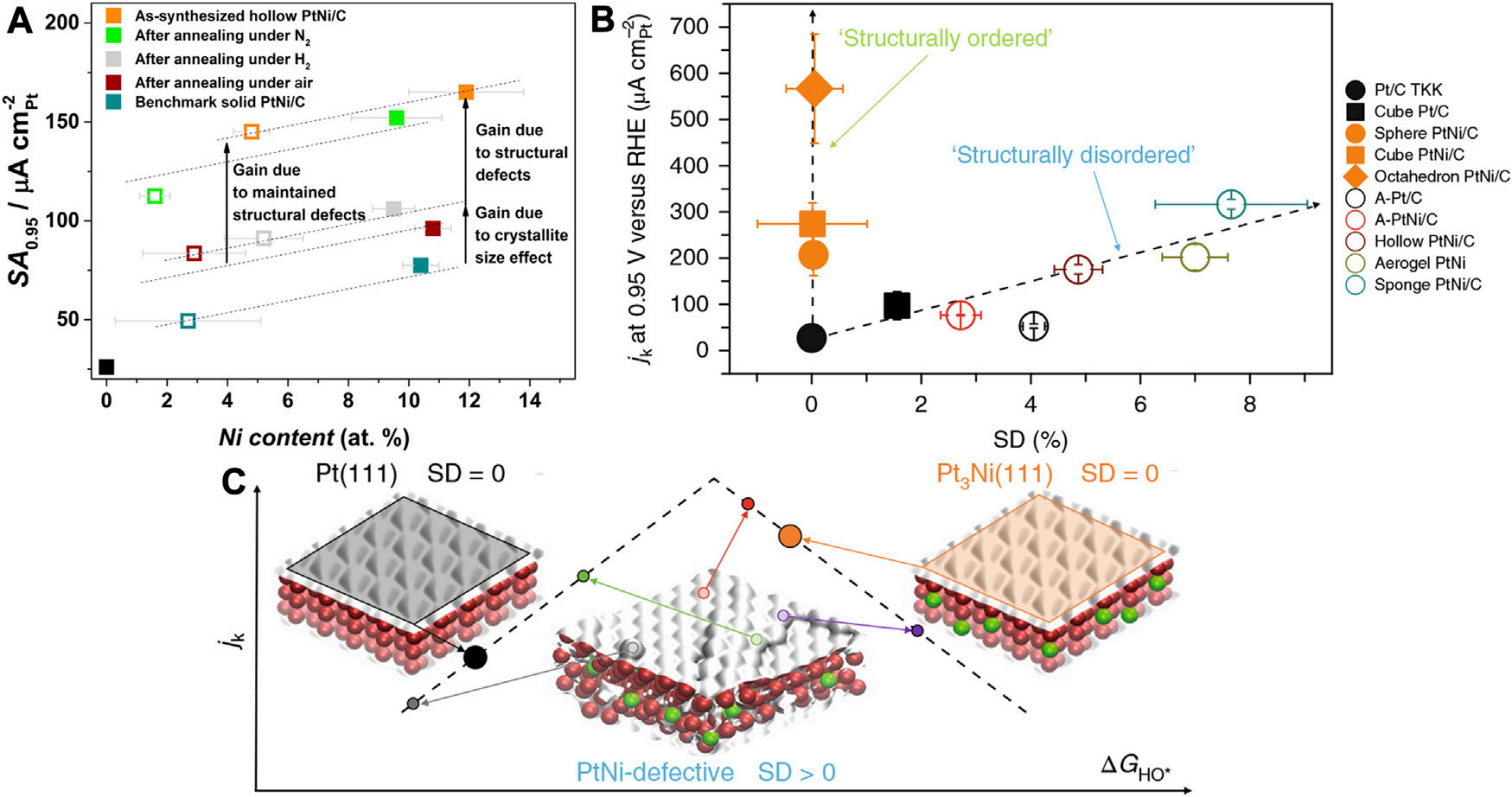
**(A)** SAs of hollow PtNi nanoparticles under different annealing atmospheres, solid and hollow squares represent the activity before and after the stability test, respectively. **(B)** Active current of nanoparticles with different SD (surface distortion). **(C)** Illustration of j_k_-∆G_HO*_ relation of two strategies to improve intrinsic activity, by homogenizing the surface and introducing defects on the surface. **(A)** Reproduced with permission (Dubau et al., 2017). Copyright 2017, American Chemical Society. **(B,C)** Reproduced with permission (Chattot et al., 2018). Copyright 2018, Springer Nature.

Maillard et al. employed wet-chemical co-reduction followed by acid etching to synthesize hollow PtNi NPs (Dubau et al., 2015). The distinct diffusion rates and etching rates of Pt and Ni lead to the formation of the nanocavity. The PtNi NPs had nanocavity, wherein the low-dimensional defects such as atomic vacancies and twin boundaries can be observed. As shown in Figure 15A, the PtNi hollow NPs are annealed in nitrogen, air, and hydrogen atmospheres to regulate the defect densities. They find that annealing in hydrogen and air results in the disappearance of the cavity and a sharp decrease of activity (Dubau et al., 2017). The elemental mapping reveals an unchanged Pt/Ni ratio after annealed treatments in different atmospheres. Synchrotron-based wide-angle X-ray scattering (WAXS) analysis suggests that upon annealing in hydrogen and air, the micro-strain of the PtNi was drastically attenuated, whereby the micro-strain can be a structural descriptor to evaluate the electrochemical activity. It is a structural descriptor, instead of an electronic descriptor like adsorption energy and overpotential.

Besides, Chattot and Maillard et al. synthesize a series of PtNi NPs with various morphologies, e.g., defect-rich (structurally disordered) NPs and defect-free single crystal (structurally ordered) NPs (Chattot et al., 2018). The micro-strain of the catalysts powder was quantified by the synchrotron-based XRD, based on which the distortion ratio of surface atoms, named as the surface distortion (SD), was calculated via the Montejano-Carrizales method (Chattot et al., 2018). As shown in Figures 15B,C, it was found that the intrinsic activity of NPs was correlated with the SD. The structurally ordered catalysts showed dramatically different intrinsic activities due to the exposure of different crystallographic facets. The Pt (111) and Pt_3_Ni (111) crystallographic facets are generally considered as highly active surfaces with a zero SD as indicated on the activity vs. ∆G_HO*_ diagram (Stamenkovic et al., 2007). However, the active sites on the disordered surface were found to be uniformly distributed with high and low activities on the diagram. Consequently, defect engineering serves as an effective strategy to optimize the intrinsic activity of the surface. A surface with various types of defects may display a competitive or ever surpassing activity compared with the structurally ordered crystallographic facets. The nanocrystal surface was usually intentionally designed into a homogeneous structure such as the sharp facets via the facet engineering as mentioned in the previous sections, but one can be inspired from this work that the same effect could be achieved by introducing defects into the surface. The facet engineering and defect engineering may seem to be the opposite design philosophy, but they could lead to the same effect by regulation on the electronic structure and hence can both improve the intrinsic activity. Therefore, defect engineering opens up a new possibility and enables extensive new methodologies toward the development of high-performance ORR electrocatalysts. Generally, the methods to generate defects include top-down etching, dealloying, and surface reconstruction by annealing, etc.

**FIGURE 16:**
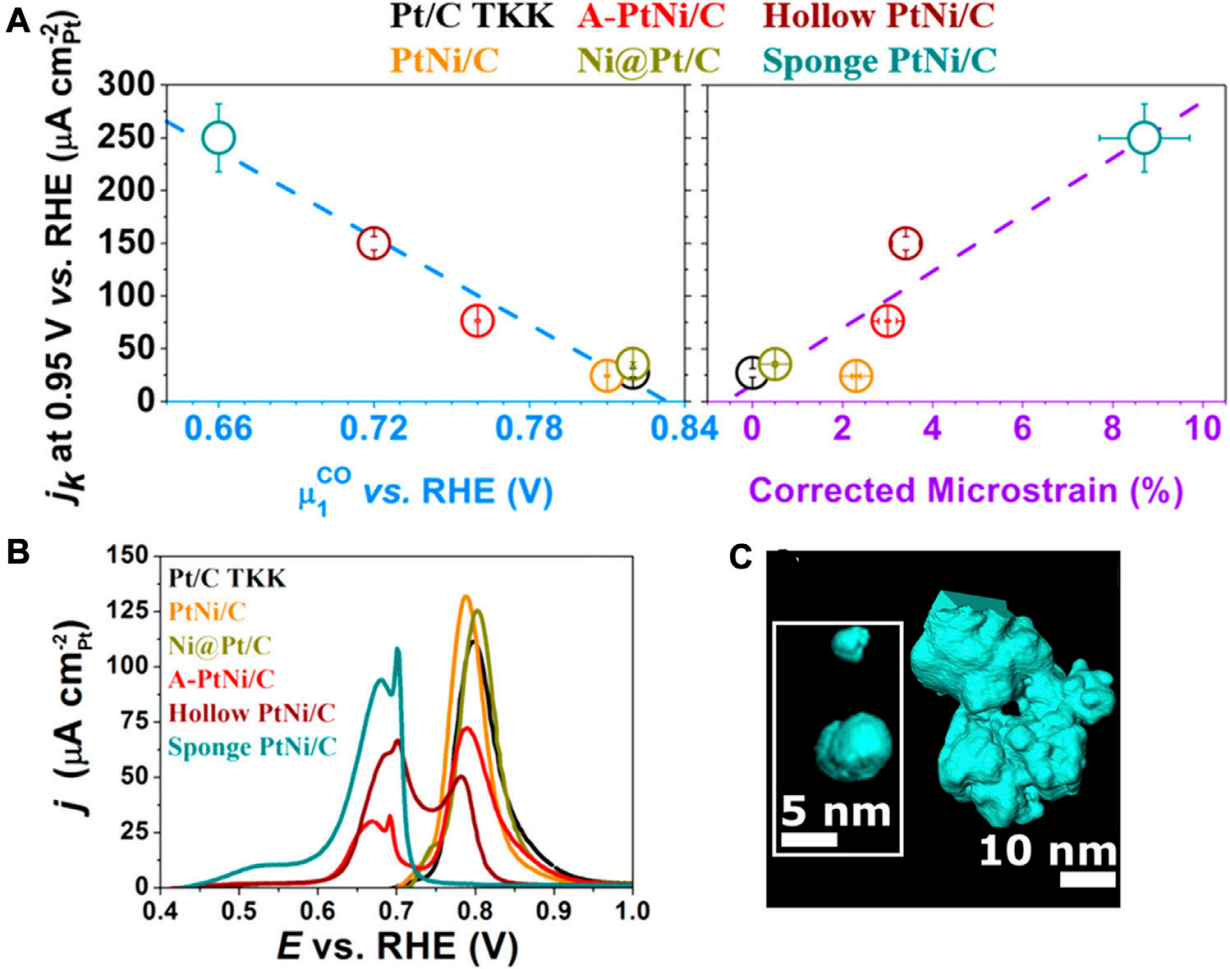
**(A)** Relationship between the CO average oxidation potential, SD, and ORR intrinsic active current. **(B)** CO oxidation curves of different nanoparticles. **(C)** Tomography simulation of Pt/C nanoparticles. **(A,B)** Reproduced with permission (Chattot et al., 2017). Copyright 2017, American Chemical Society. **(C)** Reproduced with permission (Fichtner et al., 2020). Copyright 2020, American Chemical Society.

The adsorption of CO is highly sensitive to the surface structure, so the stripping of adsorbing CO is used as a general probe for the surface local atomic structure. The surface composition, particle size, particle agglomeration, and crystallographic facets of the catalyst will affect the CO stripping profile. In the CO stripping profile, the defect-free Pt/C shows an oxidation peak at 0.7–1.0 V, while the hollow PtNi NP catalyst displays a peak at 0.4–0.73 V in addition to the normal oxidation peak (Chattot et al., 2017). The peak at 0.4–0.73 V and 0.7–1 V is speculated to be related to the CO electro-oxidation at grain boundaries, and the CO stripping from the crystalline grain respectively. The oxidation peak at 0.4–0.73 V indicates enriched crystallite boundaries at the surface of hollow PtNi, consistent with the high-resolution electron microscopic observation. As shown in Figures 16A,B, the average oxidation potential of CO, calculated based on the integration of the oxidation peaks, is found to be correlated with the surface micro-strain values determined by synchrotron-based WAXS, whereby a positive correlation of the “average oxidation potential”—“ORR intrinsic active current”—“surface distortion” is verified (Chattot et al., 2017).

Some novel defect engineering strategies have been developed for the Pt/C catalyst. Aliaksandr et al. synthesized polycrystalline Pt/C catalyst with highly defective structures through an electrochemical method (Fichtner et al., 2020). Alternating voltages were applied between two Pt wire electrodes to generate the dissolution and re-deposition of Pt. The dissolved Pt^2+^ can re-deposit to form Pt nanoparticles on the carbon black particles dispersed in the electrolyte to form Pt/C with enriched concave surface profiles with abundant defects. As shown in Figure 16C, the STEM-HAADF tomography simulation of Pt/C NPs displayed abundant steps and vacancies. The electrochemical CO stripping peak of the as-prepared Pt/C catalyst showed a negative shift, with the ORR mass activity improved by almost 2 times.

Recently, intensive efforts have been dedicated to designing highly active electrocatalysts through surface defect engineering. Nanowire is a one-dimensional nanostructure with special advantages. It has an ultra-high curved shape, and the nanowires have great potential in electrocatalysis.

**FIGURE 17:**
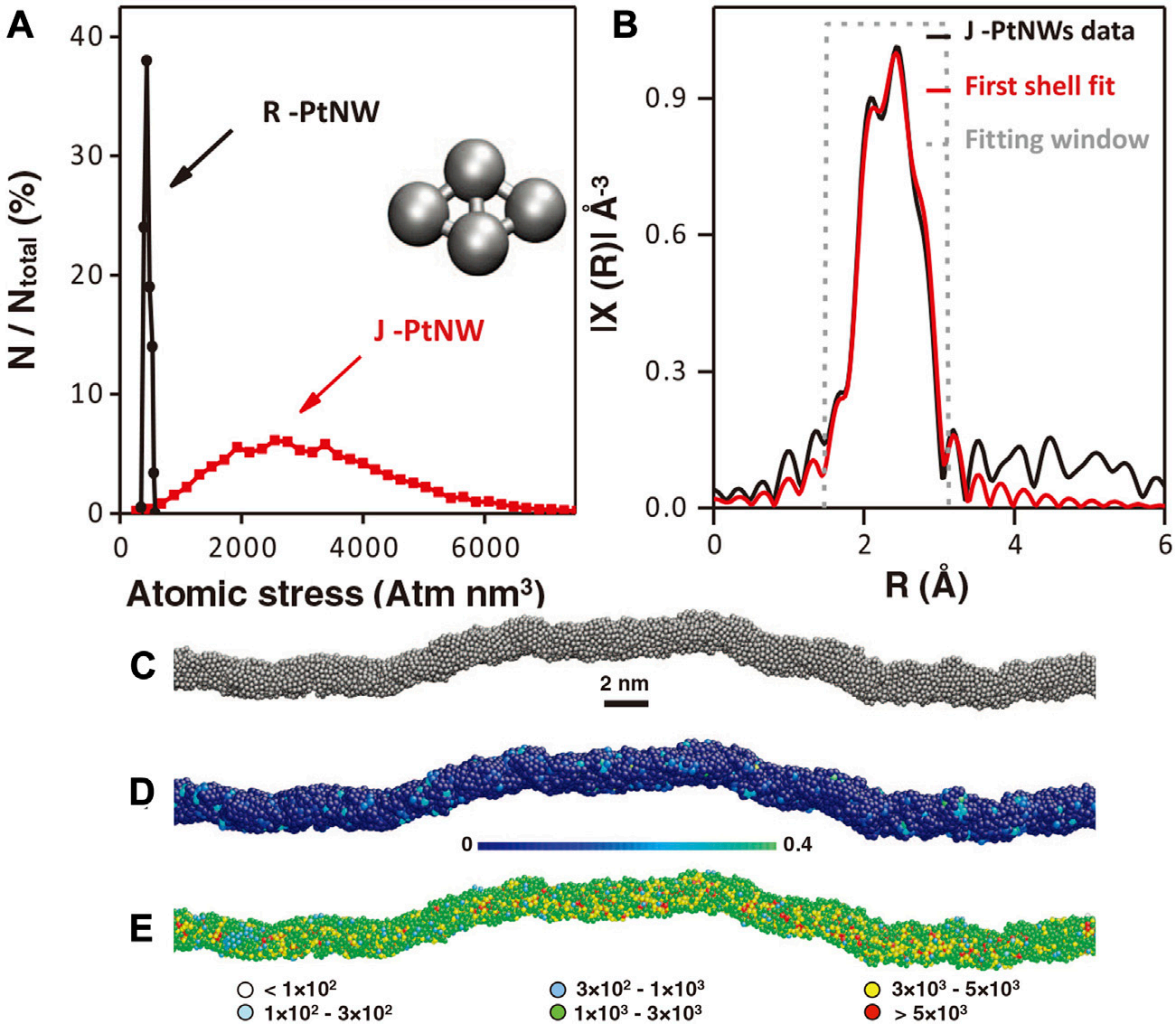
**(A)** Distribution of absolute value of average atomic stress on Pt NWs surface rhombus structure. **(B)** Pt-L_3_ edge EXAFS of J-Pt NWs. **(C)** The simulated J-Pt NWs structure through molecular dynamics simulations. **(D)** J-PtNW with colored atoms to show the five-fold index. **(E)** Different colored atoms show different absolute values of local stress in J-Pt NWs. Reproduced with permission (Li et al., 2016). Copyright 2016, American Association for the Advancement of Science.

Huang et al. demonstrated the essential role of defects by designing ultrafine Pt nanowires (Li et al., 2016). The researchers used an ingenious way to synthesize pure Pt nanowires. They first synthesized Pt@NiO core@shell structure nanowires which were then converted into PtNi alloy nanowires through thermal annealing in H_2_ atmosphere and finally obtained jagged Pt nanowires (J-Pt NWs) through electrochemical dealloying. With the continuous cyclic voltammetric polarization, the electrochemically active area was found to increase and finally approached almost 120 m^2^/g_Pt_. The authors proved that many atom vacancies, terraces, twins, and other micro-defect structures exist in the J-Pt nanowires. A record of intrinsic activity can be obtained from the J-Pt NWs to be 11.5 A/mg_Pt_. As shown in Figure 17, the reactive molecular dynamics (RMD) using the reactive force field (ReaxFF) was employed to simulate the J-Pt NW with an average diameter of ∼2.2 nm and an average length of ∼46 nm, which considered the striction regions, bending points, and jagged surfaces. The crystal-like rhombohedral surface under stress and insufficient coordination can significantly reduce the reaction barriers of the rate-determining step of ORR. The surface rhombus composed of four atoms could be arranged into two equilateral triangles, which was similar to the triangular mosaic structure on the fcc (111) surface. The rhombohedral surface on fcc (111) was found to show higher activity than on fcc (100). The extended X-ray absorption fine structure (EXAFS) results revealed that the average Pt-Pt bond length (2.71 Å) in J-Pt NWs was shrinking by 1.8% compared with that of Pt foil (2.76 Å), indicating the presence of local compressive micro-strains. Such defects are considered to be the key reason for the increased activity of J-Pt NWs.

**FIGURE 18:**
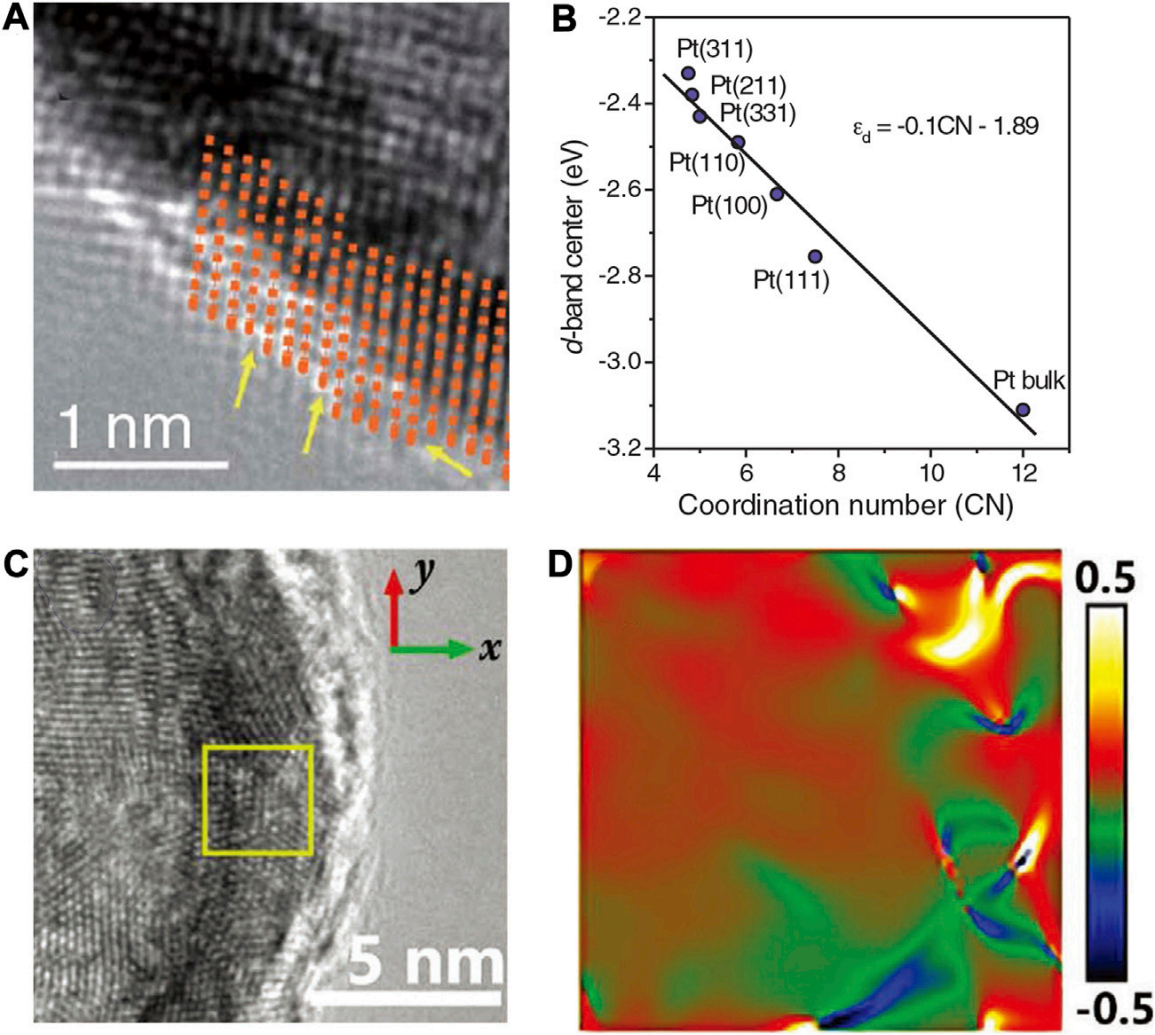
**(A)** HAADF STEM image of PtNi nano-cages. Yellow arrows mark defects such as atomic vacancies. **(B)** The DFT-derived relationship between d-band center and generalized coordination number. **(C)** HRTEM image of PdAu hollow nanochains. **(D)** The corresponding strain distribution map by GPA algorithm. **(A,B)** Reproduced with permission (Tian et al., 2019). Copyright 2019, American Association for the Advancement of Science. **(C,D)** Reproduced with permission (Jiao et al., 2020). Copyright 2020, Wiley-VCH.

Xia et al. obtain necklace-like PtNi nanowires by leaching Ni atoms with acid washing methods (Tian et al., 2019). As shown in Figure 18A by adjusting the near-surface structure and composition of the PtNi nanowires, they obtained a series of one-dimensional structures with bunched PtNi alloy nanocages, named PtNi-BNSs/C. Due to excessive Ni dissolution and collapse of the structure, a large number of defect sites can be generated in the PtNi nanocage structure, resulting in high activity. The DFT calculations revealed that the synergistic effect of strain and coordination environment related to the enriched defects, along with the incorporation of Ni and the appropriate Pt/Ni ratio, lead to a weakened Pt-O binding strength, thus boosting the intrinsic ORR activity.

The introduction of surface defects could produce micro-strain and change the d-band center. Besides, the GCN of Pt can also affect the d-band center, as shown in Figure 18B. As the commonly accepted activity descriptor for the ORR, the atomic oxygen adsorption energy 0.2 eV lower than on Pt (111) was considered as the optimal value (Luo and Guo, 2017). The DFT showed that the oxygen adsorption energy on Pt_4_Ni and Pt_3_Ni are 0.17 and 0.11 eV lower than on Pt (111), respectively. It was also found that the higher binding energy on the surface Pt atoms in Pt_4_Ni-skin may also explain the high stability of the PtNi-BNSs/C catalyst with only slight decay in activity after 50 k cycles because the defects with high surface energy were enclosed in the nano-cages. Similar to this unique hollow nanochain structure, Che et al. obtained 1D Pd-Au hollow nanochains with an ultrathin Pd-rich skin through displacement reaction (Jiao et al., 2020). The PdAu hollow nanochains (HCs) possessed periodic concave structures that displayed boosted ORR performance. These structures can readily form a high density of high-index facets. As shown in Figures 18C,D, by the Fourier transform and inverse Fourier transform of HRTEM lattice images, they calculated the strain distribution maps from HRTEM lattice images. The concave structures can deliver strong strain effects and thus caused surface charge accumulation effects. The inclusion of Au induced tensile strain in the Pd lattice. Due to the distortion of the Pd lattice, tension-compression dislocation dipoles were generated in the grade point average (GPA) strain diagrams. Compared with uncurved surfaces, the curved surface possessed an inherent strain, which was affected by the curvature on the surface. Therefore, the micro-strain was found to affect the surface electron structure and to modulate the ORR activity. Meanwhile, the one-dimensional hollow structure can also increase the conductivity and the specific surface area.

As shown in Table 4, we can see that defect engineering has become an effective strategy to regulate catalytic activity. Generally, defects can be introduced by dealloying or constructing irregularly shaped NPs, e.g., the hollow structure. More importantly, defects require reliable characterization techniques. Micro-strain, correlated to the defect, can usually be characterized in the broadening of XRD diffraction peaks. The quantitative value of micro-strain can be obtained by subtracting the effects of instrument broadening and grain size (Chattot et al., 2018). In addition, defects can also be visualized and analyzed by HRTEM(Fichtner et al., 2020) and three-dimensional STEM tomography (Pfisterer et al., 2019; Fichtner et al., 2020). The positron annihilation technique is also found to be an effective method for quantitatively investigate the defects in materials. The annihilation of positrons in a perfect lattice is usually called the annihilation of the free-state. Upon the generation of defects in the solid lattice, such as vacancies, dislocations, etc., positrons can be easily captured by these defects and annihilated, which is called positron capture annihilation, in contrast to the annihilation of free-state positrons (Dryzek and Siemek, 2019). Neutron diffraction technology can also be used to determine the micro-strain. By analyzing the displacement, broadening, and asymmetry of diffraction peaks, the twin stacking fault probability, dislocation density, and stacking fault energy can be obtained (Chen and Wang, 2016). Besides, the synchrotron-based XAFS can analyze the micro-strain by comparing the atomic bond length of the catalyst and the pure metal foils. Meanwhile, owing to the high sensitivity of the electrochemical probe to the surface structure, some electrochemical fingerprint techniques can be used to investigate the surface defects for certain material systems, such as CO stripping and CV techniques (Chattot et al., 2019).

**TABLE 4:** Examples of studies that improve ORR activity by surface defects engineering.

Catalyst	Electrolyte	SA (mA/cm2Pt + Pd)	MA (A/mgPt + Pd)	ECSA (m2/gPt + Pd)	References
PtNi nanoskeleton	0.1 M HClO_4_	2.7	1.57	58	Oh et al. (2015)
Porous PtNi NPs	0.1 M HClO_4_	1.0	0.76	76	Huang et al. (2013)
Five-fold-twinned PtCu nanoframes	0.1 M KOH	1.71	0.22	13	Zhang et al. (2016)
Porous PtCuBiMn nanosheet	0.1 M HClO_4_	2.4	0.8	33	Ud Din et al. (2017)
Octapod PtCu	0.1 M HClO_4_	—	3.2	—	Luo et al. (2017)
PtPd nanoring	0.1 M HClO_4_	4.8	3.5	73	Sun et al. (2018)
Porous PtCu NPs	0.1 M HClO_4_	1.2	0.4	33	Wang et al. (2012)
Branched PdCuCo	0.1 M KOH	0.9	0.39	43	Pingel et al. (2018)
Jagged Pt nanowires	0.1 M HClO_4_	11.5	13.6	118	Li et al. (2016)
Bunched PtNi nanocages	0.1 M HClO_4_	5.1	3.5	69	Tian et al. (2019)
Hollow PtNi	0.1 M HCl O_4_	1.3	0.56	43	Dubau et al. (2015)
PdFe nano flowers	0.1 M KOH	2.8	0.98	35	Lian et al. (2018)
Porous PdCuCo	0.1 M HClO_4_	0.25	0.18	72	Zuo et al. (2018)
Hollow PdAu	0.1 M KOH	0.14	0.33	24	Jiao et al. (2020)
Porous W-PtCuNi NPs	0.1 M HCl O_4_	2.9 ± 0.5	2.8 ± 0.5	126	Tu et al. (2019)

Nanoframes are the most common highly defective structures with high specific surface area and atomic availability. With abundant defects at the edges of the nanoframes, Yang et al. have demonstrated that Pt_3_Ni nanoframes have high ORR activity (Chen et al., 2014). Besides, they had demonstrated that Pt atoms would segregated in PtNi nanoparticles (Niu et al., 2016). The Pt atoms migrated to 24 edges to form Pt frames, which surrounded the internal Ni phase. Afterward, the etching process removed the Ni element to form highly defective Pt_3_Ni nanoframes. The method needs to synthesize nanoparticles first, and then use segregation and etching to realize the preparation of nanoframes (Bahadori et al., 2020). In addition, it can also be obtained by template method and replacement method (Nosheen et al., 2019).

However, the local physicochemical state of different defects varies a lot, meaning that not all defects can enhance the activity. The common methodology to quantify the activity in the laboratory relies on the measurement of the average kinetic current on the RDE, which usually ignores the distinctive characteristic responses of different types of defective sites on the surface. Therefore, a more accurate means to characterize the micro-defects is needed. The *in-situ* electrochemical cell-scanning tunnel microscopic (STM) technique has been employed to detect the single-site activity (Pfisterer et al., 2019). However, limited by the resolution and the interference of the STM needle itself on the catalytic reaction, this technique is still under development. High-resolution microscopic and spectrometric characterization techniques will enable accurate identification of defect sites and deeper understanding of catalytic reactions.

## Conclusion and Outlook

Electrocatalysis is known as the structure-sensitive process, whereby the activity of the electrocatalysts is mainly related to the local electronic structures. The surface electronic structures are related to the local atomic environment, which mainly refers to the atomic arrangement. From the atomic arrangement perspective, this review summarized the recent development in structural engineering from the macro-scale down to sub-nanoscale for PGM nanocatalyst, which can be classified into four strategies, to regulate ORR performance. Although these strategies may seem completely different, they are all arising from the optimization of surface interatomic distances and coordination number from the structural point of view.

The atoms on different crystallographic facets are arranged into characteristic patterns, featuring various step densities, CNs, and kinked atoms that display significant impacts on electrocatalytic activity. The structure effect of crystallographic facet lays the basis for the crystallographic facet engineering strategy. The arrangement of surface atoms is also affected by stacking modes in bulk phases. In addition to thermodynamically stable conventional crystal phases, PGM nanocatalysts with unconventional crystal phases often exhibit both excellent activity and stability, stimulating the interests in phase engineering as a design strategy. The two strategies both rely heavily on the controlled chemical synthesis of the nanocrystals.

The surface atomic distance, represented by strain, influences the interatomic electronic interaction and hence affects the surface d-band electronic structure. Therefore, strain engineering has recently been demonstrated as an effective method to regulate intrinsic activity. At an even lower length scale, the defect signifies the interruption of the periodic lattice structure, which causes a redistribution of the chemical bonding and electronic structure and hence regulates the intrinsic activity. Defect engineering has been proven to be an effective strategy with the most extensive technological methods and flexibility. These two strategies may rely mainly on the post-synthesis techniques to regulate the surface or near-surface structures.

Although significant progress has been made in the past few years, it is still difficult to design PGM electrocatalysts with high activity, high stability, and low cost. Therefore, further challenges and opportunities should be discerned in the future.

***A more precise and elaborated understanding on the structure-activity relationship should be targeted.*** It requires a combination of more precise *in-situ* characterization techniques and reliable analyses (Li et al., 2019b). Generally, the characterization of nanoscale materials follows a certain logic. For instance, the atomic arrangement can be obtained by X-ray diffraction techniques, to correlate the crystallographic facets index, crystal phase, and performance. Besides, the lattice strain is usually manifested as a shift in the diffraction peak in the XRD patterns. Defects usually cause localized lattice distortion, which includes local tensile and compressive strain. Consequently, defects are often manifested as widening of diffraction peaks in the XRD patterns. Furthermore, the subtle characterization of chemical environments relies on the synchrotron-based XAFS. Besides, catalytic performance is typically measured and evaluated by RDE or membrane electrode assembly (MEA), but they normally reflect the average activity of a large number of particles in a statistical sense. Therefore, a more accurate and *in-situ* characterization of a single catalyst particle or even active site may better elaborate a more precise structure-activity relation. The newly developed identical location TEM technology or *in-situ* environmental TEM technology based on electron microscopic technique could be options to fulfill the target. In addition, *in-situ* XPS is used to detect the electronic structure evolution of the active site, and *in-situ* FTIR can detect intermediate substances (Dong et al., 2019).

***It is necessary to combine theory and experiment to develop more mature theoretical models.*** The theory of adsorption energy has been used for many model catalysts (Seh et al., 2017). However, the real catalysts are complicated, leading to big differences between actual catalysts and simplified models used in DFT. Besides, the scaling relationship in adsorption energy theory shows the limitation in optimizing the d-band center on the volcano plot. Recently, Liu et al. investigated the elementary step energy barrier corresponding to kinetics characteristics in detail (Tao et al., 2019). Based on ORR kinetic fingerprint, they constructed a quantitative relationship between the reaction potential energy and activity. The kinetic study of the catalysts could identify the rate-limiting steps and better instruct the optimization efforts toward better catalytic performance, which may serve as a more reasonable guideline to the design of the structure of a constructive catalyst (Seh et al., 2017; Sakaushi et al., 2018; Tao et al., 2019).

***It is necessary to focus on the interface effect between the noble metal and the carrier.*** The electron interaction between the carrier and the metal catalyst can strongly affect the activity of the catalytic site (Chang et al., 2020). Dong et al. confirmed the electron transfer behavior between Pd and Mo_2_C substrates, and found that the covalent interaction between the Pd catalyst and the carbide support can reduce the d band center position of Pd (Huang et al., 2021). In addition to the carbon-based support, metal oxides can form heterojunctions with noble metal catalysts, which can not only achieve interface electronic effect, but also achieve synergistic effect. Peng et al. developed SnO_x_/Pt-Cu-Ni heterojunction catalyst (Shen et al., 2019). The SnO_x_ support could promote the occurrence of the first two primitive reactions of ORR, while the remaining steps occurred at the Pt-Cu-Ni site. They showed this dual-site cascade mechanism by SnO_x_/Pt-Cu-Ni heterojunction. In addition, the confinement effect of the carrier can slow down the corrosion rate of the noble metal catalyst and improve the stability of the catalyst (Gerber and Serp, 2019). Therefore, it is of great significance to study the interface effect between the noble metal and the carrier.
